# Changes in Metabolism and Proteostasis Drive Aging Phenotype in *Aplysia californica* Sensory Neurons

**DOI:** 10.3389/fnagi.2020.573764

**Published:** 2020-09-15

**Authors:** Nicholas S. Kron, Michael C. Schmale, Lynne A. Fieber

**Affiliations:** Department of Marine Biology and Ecology, Rosenstiel School of Marine and Atmospheric Science, University of Miami, Miami, FL, United States

**Keywords:** transcriptomics, time series, buccal ganglion, pleural ganglion, survival curve

## Abstract

Aging is associated with cognitive declines that originate in impairments of function in the neurons that make up the nervous system. The marine mollusk *Aplysia californica* (Aplysia) is a premier model for the nervous system uniquely suited to investigation of neuronal aging due to uniquely identifiable neurons and molecular techniques available in this model. This study describes the molecular processes associated with aging in two populations of sensory neurons in Aplysia by applying RNA sequencing technology across the aging process (age 6–12 months). Differentially expressed genes clustered into four to five coherent expression patterns across the aging time series in the two neuron populations. Enrichment analysis of functional annotations in these neuron clusters revealed decreased expression of pathways involved in energy metabolism and neuronal signaling, suggesting that metabolic and signaling pathways are intertwined. Furthermore, increased expression of pathways involved in protein processing and translation suggests that proteostatic stress also occurs in aging. Temporal overlap of enrichment for energy metabolism, proteostasis, and neuronal function suggests that cognitive impairments observed in advanced age result from the ramifications of broad declines in energy metabolism.

## Introduction

Aging can be summarized as a progressive decline in the physiological function and increased vulnerability to death of an organism over the course of the lifespan (Lopez-Otin et al., 2013; Dodig et al., 2019). Aging is a prime risk factor for many of the most common and deadly diseases, including heart disease, diabetes, and cancer (Franceschi et al., 2018). Several of the most prevalent neurological diseases, such as Alzheimer’s disease and Parkinson’s disease, have age-associated onset (Hou et al., 2019). Even non-pathological aging is associated with a slew of metabolic and cognitive changes that have broad impacts on public health. The nervous system is profoundly affected with age, resulting in cognitive impairments and susceptibility to neurodegenerative disorders (Reuter-Lorenz, 2002; Yankner et al., 2008; Anderson and Craik, 2017; Bettio et al., 2017). The long-lived, post-mitotic, and energetically expensive nature of neurons results in a suite of age-associated changes that underpin cognitive changes observed at the whole organism level (Grimm and Eckert, 2017).

Metabolic drift and broad scale decreases in aerobic glycolysis, the tricarboxylic acid cycle, and oxidative phosphorylation are a common feature of brain aging (Pandya et al., 2015, 2016; Ivanisevic et al., 2016; Pollard et al., 2016; Goyal et al., 2017; Mattson and Arumugam, 2018; Watts et al., 2018; Guo et al., 2020). Metabolic impairments are associated with dysfunctional mitochondria and increasing levels of reactive oxygen species (ROS), which damage proteins and membranes (Di Domenico et al., 2010; Paradies et al., 2011; Diot et al., 2016; Islam, 2017). Increased iron toxicity and the accumulation of ectopic fat deposits in neurons are common indicators of an increasingly oxidative state in aging (Ward et al., 2014; Palikaras et al., 2017; Jin et al., 2018). These changes are believed to contribute to decreased protein homeostasis and chronic inflammation (Hohn et al., 2013; Barrientos et al., 2015; Currais, 2015; Hohn et al., 2017; Martinez et al., 2017; Garaschuk et al., 2018; Mattson and Arumugam, 2018; Yang et al., 2019).

Dysfunction of the endoplasmic reticulum and mitochondria results in disrupted calcium dynamics and various signaling pathways crucial to neuronal function (Krebs et al., 2015; Pandya et al., 2015; Marchi et al., 2018). Disruptions of synaptic plasticity as a result of impaired signaling drive cognitive declines typical of brain aging (Magnusson et al., 2010; Haxaire et al., 2012; Morrison and Baxter, 2012). While these neuronal signatures are known to occur even in non-pathological aging, they are also typical of major neurodegenerative disorders such as Parkinson’s disease and Alzheimer’s disease (Silvestri and Camaschella, 2008; Thomsen et al., 2015; Foster et al., 2017; Grimm and Eckert, 2017; Kimura et al., 2017).

Among the most popular models for the study of aging in the nervous system are mammalian models such as the lab mouse *Mus musculus* and the lab rat *Rattus norvegicus*, invertebrate models such as the nematode worm *Caenorhabditis elegans* and fruit fly *Drosophila melanogaster*, as well as the budding yeast *Saccharomyces cerevisiae* (Mayer and Baker, 1985; Weindruch and Masoro, 1991; Olsen et al., 2006; Terzibasi et al., 2007; Sundberg et al., 2011; Lippuner et al., 2014). Comparisons among these diverse models and with humans have elucidated many of the common processes of aging among metazoans, including those in the nervous system. Discoveries in invertebrate models *C. elegans* and *D. melanogaster* in particular have contributed significantly to furthering the understanding of aging at a molecular level (Stegeman and Weake, 2017). However, these popular invertebrate aging models represent only the ecdysozoan clade of invertebrates.

As a representative of the lophotrochozoan phylum Mollusca, the marine gastropod model, the California sea hare *Aplysia californica* (Aplysia) broadens understanding of aging in metazoans. Due to a relatively simple nervous system made up of gigantic neurons, Aplysia is a well-studied model for the nervous system (Moroz, 2011). This, in addition to a 1-year lifespan, makes Aplysia an ideal model for studying the effects of age on the nervous system (Rattan and Peretz, 1981; Bailey et al., 1983; Peretz et al., 1984; Peretz and Srivatsan, 1992; Kempsell and Fieber, 2014). Furthermore, phylogenetic analysis has demonstrated that Aplysia represents an evolutionarily closer model to vertebrates than currently used ecdysozoan models, suggesting that Aplysia may offer a more effective model for human neuronal aging than do ecdysozoans (Moroz et al., 2006). As with *Drosophila* and *C. elegans*, a reference genome and transcriptome are available for Aplysia (GCF_000002075.1), which has allowed for investigation of transcriptional changes in aging Aplysia neurons; however, there is a distinct absence of transcriptional profiling across the aging process as has been done in other models (Moroz and Kohn, 2010; Kadakkuzha et al., 2013; Moroz and Kohn, 2013; Greer et al., 2018). Sampling of multiple time points is imperative for a full understanding of the transcriptional dynamics of aging and addressing which phenomena may be drivers of age-related dysfunctions.

To fill this gap and better understand the behavioral and physiological changes known to occur in this species with age, we performed a transcriptional time series experiment in Aplysia sensory neurons (SN). Specifically, we selected the buccal S (BSC) and pleural ventral caudal (PVC) SN, which comprise a small number of mostly homogeneous neurons (roughly 150 and 200, respectively) that can be reliably identified and sampled between individuals (Walters et al., 2004; Fieber et al., 2013). Age-associated declines in excitability of BSC are suggested to contribute to impairments of the biting reflex, likely reflecting the role of BSC in slowed feeding and declines in mass in Aplysia of advanced age (Fieber et al., 2010; Kempsell and Fieber, 2014). Similar physiological changes in PVC contribute to decreased sensitization of the sensory-motor synapse of the tail withdrawal reflex (TWR), which, like the biting reflex, is also compromised in aging (Kempsell and Fieber, 2014, 2015b). This degree of precision and the vertical integration that is possible throughout the aging process using behavioral, physiological, and now molecular data involving these discrete groups of neurons is perhaps unique to this animal model.

## Materials and Methods

### Animal Rearing

Two hundred individuals from a single egg mass (cohort) of *A. californica* were reared at the University of Miami National Resource for Aplysia under standard hatchery conditions as described previously (Gerdes and Fieber, 2006). Animals were fed an *ad libitum* diet of *Aghardiella subulata* and stocked at a density of two to seven animals per 16-L cage. Half of all individuals in cages were weighed monthly to assess cohort growth. The natural mortality of 53 individuals was recorded to monitor the aging rate of the cohort. To estimate the aging rate of the cohort, the Gompertz survivorship function $s=\exp\left[\left(\frac{A}{G}\right)\,\left(1-e^{G\,t}\right)\right]$ was fit to the mortality data, where *A* is the initial mortality rate and *G* is the actuarial aging rate, as done previously (Wilson, 1994; Kempsell and Fieber, 2014).

### Behavior Assessment and Sampling

Immediately prior to sexual maturity (approximately 6 months of age) and monthly thereafter, a minimum of 12 individuals were selected for behavioral assessment using a random number generator (Table 1). Animals were weighed and then placed in solo cages to acclimate overnight. The next morning, reflex performance was assessed in each individual based on time to right (TTR) behavior and time to relax the tail following tail pinch, called the tail withdrawal reflex (TWR), as described previously (Greer et al., 2018). Each reflex behavior was assessed in triplicate, with a minimum of 5 min rest between replicates and 15 min rest between TTR and TWR. Reflex data were analyzed with a Kruskal–Wallis and pairwise Wilcoxon *post hoc* test with Benjamini–Hochberg false discovery rate correction. Animals were then housed in solo cages and fasted for 2 days after behavioral assessment before sacrifice and sampling to prevent behavioral assessment from biasing expression in target neurons as described previously (Greer et al., 2018).

**TABLE 1:** Number of individuals used for each step of the experimental process.

Age (months)	*n* behavior	Tissue	*n* samples	*n* RNA	*n* passed QC	*n* sequenced	*n* differential expression
6	12	BSC	12	10	1	0	0
		PVC	12	11	6	5	5
7	12	BSC	12	9	6	6	6
		PVC	12	10	6	6	6
8	12	BSC	12	9	6	6	6
		PVC	12	9	6	6	5
9	12	BSC	12	11	6	6	5
		PVC	12	9	6	5	5
10	14	BSC	14	12	6	6	6
		PVC	14	14	6	6	6
11	18	BSC	17	10	6	6	5
		PVC	17	12	6	6	6
12	15	BSC	13	11	6	6	6
		PVC	13	13	6	6	6
Sum	95	BSC	92	72	37	36	34
		PVC	92	78	42	40	39
		Total	184	150	79	76	73

*This table breaks down the number of animals or samples used in the major steps of the experimental pipeline, namely: behavioral analysis, tissue microdissection, RNA extraction, RNA and sequencing library quality control, sample library sequencing, and sample raw reads used for differential expression analysis. Individuals are separated into rows by sampling time: 6, 7, 8, 9, 10, 11, and 12 months post hatch. Rows are further subdivided for each sample based on the tissue of origin for the sample: buccal S cluster (BSC) or pleural ventral caudal cluster (PVC).*

Animals were prepared for sacrifice by injecting 1/6 bodyweight of cool, isosmotic MgCl_2_, with a wait of 5 min for anesthetic to take effect, as evidenced by lack of response to painful stimuli. The whole nervous system was then dissected out, euthanizing the animal. Each ganglion was washed in two separate baths of sterile artificial seawater. Target neuron clusters were microdissected from their respective hemi-ganglia after pinning in a sylgarded dish as described previously (Fieber, 2000). Samples from the same paired hemi-ganglia were pooled and stored in 0.3 ml of RNA Protect Cell Reagent (Qiagen) at −80°C.

### RNA Extraction

Total RNA was extracted from target neuron clusters using the Qiagen RNeasy Micro Kit according to manufacturer protocol. Residual genomic DNA was eliminated with a 15-min DNase incubation. RNA purity was measured using a NanoDrop 1000 spectrophotometer (Thermo-Fisher), integrity was measured with an Agilent RNA 6000 Nano kit on an Agilent 2100 Bioanalyzer, and quantity was measured with a Qubit 3.0 fluorometer using the Qubit RNA HS assay kit (Thermo-Fisher). The six highest-quality samples in terms of RNA integrity and purity were selected for further processing (Table 1).

### Library Preparation and Sequencing

Total RNA from samples was used to generate sequencing libraries with the Illumina TruSeq Stranded mRNA High-Throughput kit (150–250 ng) following manufacturer protocol. RNA was poly-A selected using poly-T oligo attached magnetic beads, after which RNA was heat fragmented for 6 min to achieve desired fragment size of between 200 and 300 base pairs. Agilent DNA 1000 kit was used to verify library fragment size on an Agilent bioanalyzer 2100. First-strand cDNA synthesis was random primed. Library concentrations were quantified using the Qubit dsDNA HS Assay kit on a Qubit 3.0 and sent to the University of California Irvine Genomics High-Throughput Facility (UCI GHTF) for further quality control and sequencing. At UCI GHTF, libraries were quantified using KAPA Library Quantification Kit (KAPA Biosystems), multiplexed, and sequenced across eight lanes of an Illumina HiSeq 2500 high-throughput sequencer as 100 base pair paired-end reads. Raw reads were deposited in the NCBI SRA and can be found under the following BioProject ID: PRJNA639857.

### Read Quality Control and Mapping

Raw read quality was assessed using the FastQC software tool^1^. The BBDuk software tool from the BBtools package was used for adapter removal, quality trimming, and length and quality filtering (Bushnell, 2014). Trimmed and filtered reads were then reassessed with FastQC before mapping to the *A. californica* reference transcriptome (AplCal3.0 GCF 000002075.1^2^) and quantification using the *Salmon* software tool (Patro et al., 2017). Transcript abundances from *Salmon* were then imported into the R statistical environment using the *tximport* R package (R Core Team, 2013; Soneson et al., 2015). A list of software tools used in this analysis are listed in Supplementary Table 1.

### Data Preparation and Visualization

Data were formatted and visualized in the R statistical environment using the *tidyverse* R package suite and the *complexHeatmap* R package (Gu et al., 2016; Wickham et al., 2019). A detailed list of all R packages and versions used can be found in the Supplementary Material. Transcript abundances in transcript per million (TPM) from *Salmon* were filtered to exclude any transcript that did not have a minimum TPM of 1 in at least one time point. Raw TPMs were log transformed by taking log base 2 of TPM + 1 for clustering and visualization. A full readout of R packages used in this study can be found in Supplementary Table 2.

### Principal Component and Surrogate Variable Analysis

Transcript abundances were centered and scaled using the scale() base R function. Principal component analysis (PCA) was performed on the top 1000 most variable transcripts with the prcomp() function from the stats R package.

Surrogate variables were identified and quantified from transcript abundances using the *sva* R package (Leek, 2014). Surrogate variables were corrected for using the removeBatchEffect() function from the *limma* R package with log-transformed TPM as input (Ritchie et al., 2015). Corrected values were only used for visualization and clustering. For differential expression analysis, surrogate variables were included as factors in the model design.

### Differential Expression Analysis

Differential expression analysis was performed at the transcript level in each tissue separately using the likelihood ratio test (LRT) from the DESeq2 R package (Love et al., 2014). LRT is particularly useful in time course experiments because it analyzes all time points at once, as opposed to pairwise comparisons between time points. This allowed the detection of transcripts that change across time points while avoiding extra multiple test correction incurred by sequential pairwise comparison of time points. Specifically, the LRT identifies significantly DE transcripts by comparing difference in deviance between a fully parameterized model and a reduced model that omits the time variable. Transcripts with a false discovery rate-corrected *p*-value (*p*_adj_) less than or equal to 0.01 were considered significantly differentially expressed.

### Expression Profile Clustering

Because LRT only identifies transcripts that vary significantly with time, differentially expressed transcripts were clustered according to their expression profiles using the DIANA clustering to identify transcripts with coherent patterns of expression across time. Clustering was performed via the function degPatterns() from the *DEGreport* R package (Pantano, 2017). The log2 expression of biological replicates within each month was averaged and then the maximum and minimum were subtracted, yielding the maximal log2 fold difference for each transcript. Only transcripts with a maximal log2 fold differences of 0.58 or more used as input for clustering.

### Gene Ontology and KEGG Analysis

The *A. californica* reference transcriptome was annotated by using the blastx function from the *BLAST* + software package against a local blast databased built from the UniProt human proteome (UP000005640), selecting only the top hit with a minimum *e*-value of 10^–3^ (Camacho et al., 2009). Aplysia proteins were mapped to human orthologs to leverage the resources available for the comparatively much better annotated and studied human proteome.

For Kyoto Encyclopedia of Genes and Genomes annotation, the Aplysia proteome was downloaded from the NCBI ftp site and annotated for KEGG Ortholog (KO) terms using the *ghostKOALA* tool from the KEGG Automatic Annotation and KEGG Mapping Service (Kanehisa et al., 2016). We chose to map to KO as opposed to the *Homo sapiens* annotation KEGG database to be able to leverage annotations of evolutionarily more proximate invertebrates, particularly mollusks. KEGG pathway enrichment was visualized with the *pathview* R package (Luo and Brouwer, 2013).

UNIPROT identifiers were used in Gene Ontology (GO) enrichment analysis for Biological Process, Cellular Compartment, and Molecular Function ontologies with the *clusterProfiler* R package (Yu et al., 2012). GO terms with hyper-geometric test adjusted *p* ≤ 0.05 for each cluster were considered significantly enriched. To aid in interpretation, GO enrichment was limited to the 4th level of the GO hierarchy. A second GO analysis was performed using the *topGO* R package due to its robust utilization of the GO graph structure. The algorithm parameter was set to “elim” and statistic parameter set to “fisher” (Alexa and Rahnenfuhrer, 2018). Terms with an enrichment *p-*value ≤ 0.01 were considered significant.

KO enrichment analysis was similarly performed with *clusterProfiler* R package (Yu et al., 2012). KEGG Orthology Pathways for each cluster with hyper-geometric test adjusted *p* ≤ 0.01 were considered significantly enriched. Enriched pathways were visualized using the *pathview* R package (Luo and Brouwer, 2013).

All code used for data preparation and analysis in this study can be found at the following GitHub repo: https://github.com/Nicholas-Kron/Kron_Cohort77_Differential_Expression_Analysis.

## Results

### Cohort Weight and Survivorship

**FIGURE 1:**
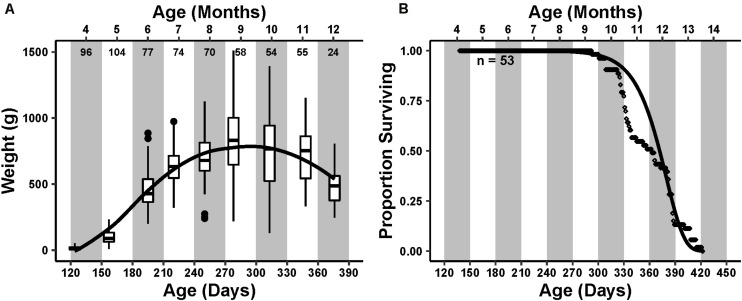
Cohort life history. Primary *x*-axis is the time in days post hatch, with the secondary *x*-axis at the top representing the age in months that is further emphasized by the alternating white and gray bars in the plot area. **(A)** Cohort weight trend. Box and whisker plots represent the interquartile range and median of weight measured at each monthly sampling point. Small numbers below the secondary axis are the number of individuals weighed that month. The curve drawn over the boxplots represents a loess smoothing of the data, showing an increasing and then decreasing weight trend peaking at age 9–10 months. **(B)** Survivorship of 53 individuals. Each point is the daily proportion of animals surviving. A Gompertz survival function, $s=\exp\left[\left(\frac{A}{G}\right)\,\left(1-e^{G\,t}\right)\right]$, was fit to the survivorship data visualized as a curve over the points. Initial mortality rate (A) and actuarial aging rate (G) were 7.4 × 10^–10^ and 0.047, respectively.

Average mass of the animals in the cohort steadily increased until reaching an inflection point during the ninth month of age, after which it decreased (Figure 1A).

The first natural mortality occurred during the ninth month of age at 293 days post hatch. The proportion of total deaths steadily increased thereafter, reaching a maximum during the 12th month of age (Figure 1B). The final mortality occurred during the 14th month of age at 422 days post hatch. The median lifespan was 363 days. The calculated Gompertz actuarial aging rate G, which describes the change in mortality rate with change in time, was 0.047, similar to previously reported aging rates (Gerdes and Fieber, 2006).

### Reflex Behaviors

**FIGURE 2:**
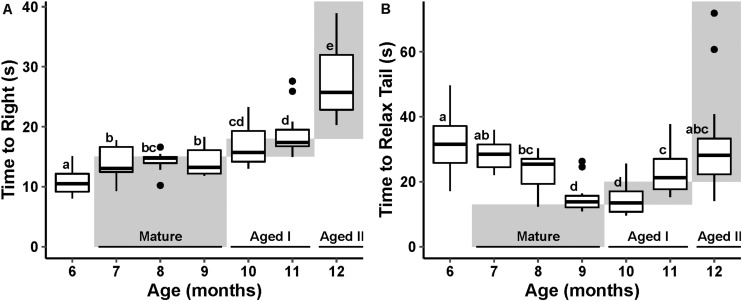
Cohort reflex behavior performance with age. The stages of aging behavior range defined by Kempsell and Fieber (2014) are represented as gray boxes in the plot space and labeled at the bottom of the plot. Boxes labeled with different letters are considered significantly different by pairwise Wilcoxon test (fdr-corrected *p* ≤ 0.05). **(A)** Boxplot of righting reflex behavior performance at each month of randomly selected animals. **(B)** Tail withdrawal reflex behavior performance at each month of randomly selected animals.

Data for both reflex behaviors failed to meet assumptions of normality and thus were tested using non-parametric tests. TTR varied significantly with age (*p* ≤ 0.01, Kruskal–Wallis, ε^2^ = 0.67). Pairwise Wilcoxon test (Benjamini–Hochberg fdr-corrected *p* ≤ 0.05) revealed a significant increase in TTR with age (Figure 2A), conforming to an expected pattern with age defined by Kempsell and Fieber (2014).

TWR also varied significantly with age (*p* ≤ 0.01, Kruskal–Wallis, ε^2^ = 0.48). Pairwise Wilcoxon test (fdr-corrected *p* ≤ 0.05) revealed a significant decrease in TWR through the Mature life stage followed by a significant increase in TWR in the Aged life stages. This U-shaped pattern with age is different from the pattern described in Kempsell and Fieber (2014; Figure 2B); however, the steady increase in time to execute TWR after age 10 months is consistent with earlier reports (Kempsell and Fieber, 2014; Greer et al., 2018).

### Principal Component and Surrogate Variable Analysis

Transcripts with zero expression across all time points and transcripts without a minimum TPM of 1 in at least one time point in one tissue were filtered out, resulting in 12,002 analysis ready transcripts.

**FIGURE 3:**
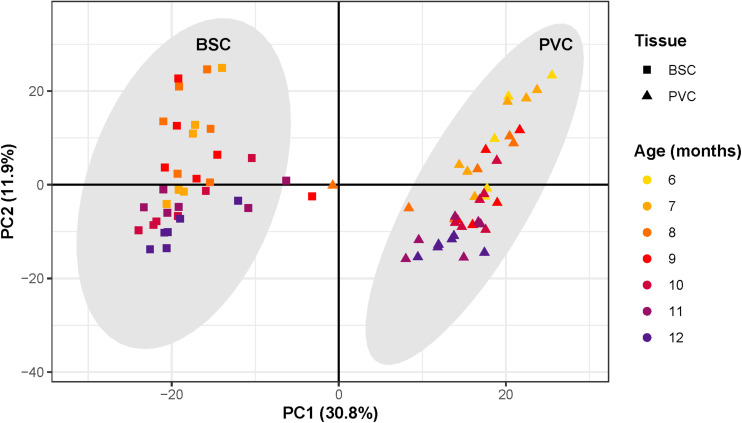
Principal component analysis of RNAseq samples. Scatter plot of principal component analysis with first principal component along the *x*-axis and second principal component along the *y*-axis. Each point corresponds to an individual sample. The shape of each point corresponds to sample tissue type, square for Buccal S Cluster (BCS) and triangles for Pleural Ventral Caudal cluster (PVC). Point color corresponds to age, from 6 to 12 months. Samples segregate along the first principal component according to tissue, forming two distinct clusters. Gray ovals around each cluster represent the cluster 99.7% confidence intervals. Three samples outside the tissue-cluster confidence intervals were considered outliers. Samples segregate roughly according to chronological age along the second principal component.

PCA revealed that the first three principal components (PCs) explained 53% of the total variance of samples. Samples segregated strongly according to tissue along PC1, which explained 31% of the total variance (Figure 3). Three samples clustered outside the 99.7% confidence intervals of each tissue-specific cluster and thus were considered as outliers and removed from all further analysis. Samples separated roughly according to chronological age along PC2, which explained 12% of the total variance.

Surrogate variable analysis identified one surrogate variable. This surrogate variable correlated strongly with PC3, which explained 10% of the total variance. The identified surrogate variable was accounted for in downstream analysis by including it in the design model. Since variance due to PC1, a proxy for tissue type, was roughly triple that of PC2, a proxy for chronological age, differential expression analysis was performed separately on each tissue to maximize signal due to aging.

### Differential Expression

LRT from the DESeq2 R package identified 1647 and 2032 differentially expressed (DE) transcripts (fdr-corrected *p* ≤ 0.01) for BSC and PVC, respectively, with 689 DE transcripts shared between the two tissues. A full list of differential expression results for both tissues can be found in Supplementary Tables 3, 4.

### Clustering

**FIGURE 4:**
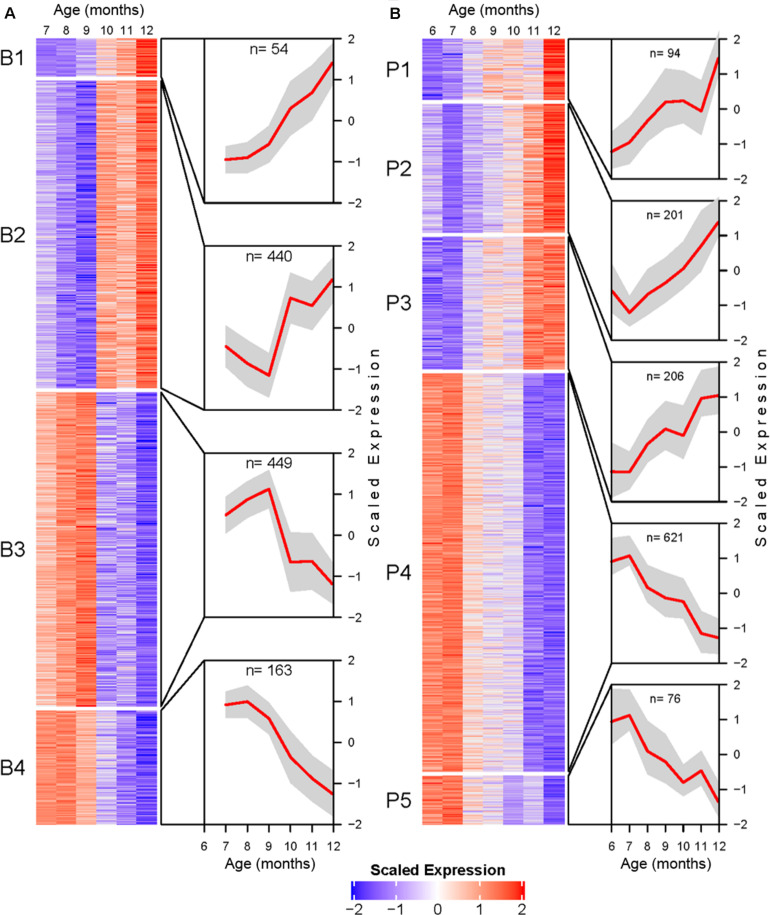
Transcriptional profile cluster heatmaps and trajectories for BSC **(A)**, and PVC **(B)** SN. Transcripts determined to be differentially expressed by likelihood ratio test in each tissue were clustered using the DIANA clustering algorithm. Each heatmap corresponds to a cluster. Heatmap cells are mean centered and variance scaled average monthly expression for each transcript (rows) at each month (columns). Cutouts illustrate the cluster average scaled expression (*y*-axis) at each month (*x*-axis) as a red line with standard deviation as a gray polygon. Total number of genes per cluster are listed at the top of each cutout.

Transcripts identified as DE were further processed for temporal profile clustering, resulting in 1319 and 1620 clustering ready transcripts for BSC and PVC, respectively. Clustering resulted in four BSC clusters and five PVC clusters (Figure 4). A full list of transcript cluster assignment results for both tissues can be found in Supplementary Tables 3, 4.

BSC clusters exhibit either increasing expression with age (B1 and B2) or decreasing expression with age (B3 and B4). The transcriptional trajectories of the two largest clusters (B2 and B3) form a mirrored pair. Cluster B2 decreases in average expression until reaching a minimum at 9 months, after which the expression trend sharply inflects and rapidly increases. Cluster B3 exhibits an inverse pattern to B2, increasing to a maximum at 9 months followed by rapidly decreasing average expression. The smaller clusters B1 and B4 are roughly monotonic in profile (Figure 4A).

PVC clusters exhibit similar general increasing or decreasing trends (Figure 4B). Roughly monotonically increasing (P2 and P3) or decreasing (P4 and P5) clusters represent the majority of DE PVC transcripts. Finally, cluster P1 shows a roughly logistic trajectory until approximately age 12 months, at which point the profile spikes.

### Transcript Annotation Rates

BLAST annotation of Aplysia transcripts to the UNIPROT human proteome resulted in roughly 70% of all Aplysia transcripts mapping to a human ortholog (Table 2). The annotation rate for KEGG was much less, approximately 45%. However, KEGG and UNIPROT annotation rates were much higher for significantly DE transcripts, roughly 60% for KEGG and 80% for UNIPROT. For transcriptional profile clusters, the KEGG annotation rate geometric mean was approximately 53%, and that of UNIPROT was approximately 75%. A full list of BLAST annotation results can be found in Supplementary Table 5. A full list of KEGG annotation results can be found in Supplementary Tables 6, 7, Supplementary Figure 1, and Supplementary Data Sheets 1, 2.

**TABLE 2:** Transcript annotation rates.

Category	Transcripts *n*	KEGG *n* (%)	UNIPROT *n* (%)
All transcripts	28,786	13,431 (46.7%)	20,580 (71.5%)
Passed Filter	12,002	6885 (57.4%)	9669 (80.6%)
BSC LRT *p*_adj_ ≤ 0.01	1647	976 (59.3%)	1328 (80.6%)
PVC LRT *p*_adj_ ≤ 0.01	2032	1236 (60.8%)	1662 (81.8%)
Cluster B1	54	22 (40.7%)	36 (66.7%)
Cluster B2	440	267 (60.7%)	354 (80.5%)
Cluster B3	449	233 (51.9%)	357 (79.5%)
Cluster B4	163	88 (54%)	118 (72.4%)
Cluster P1	94	49 (52.1%)	66 (70.2%)
Cluster P2	201	99 (49.3%)	146 (72.6%)
Cluster P3	206	120 (58.3%)	146 (70.9%)
Cluster P4	621	403 (64.9%)	539 (86.8%)
Cluster P5	76	39 (51.3%)	64 (84.2%)
	Clusters Geometric Mean:	53.3%	75.7%

*The total number of transcripts is reported for all transcripts listed in the AplCal3.0 gene feature format file, the number that passed data filtration, the number tested significant by likelihood ratio test (LRT) in buccal S cluster (BSC) and pleural ventral caudal cluster (PVC) neurons, and the total number of transcripts in each transcriptional profile cluster. For each category, the number of transcripts that were annotated to a human UNIPROT identifier by blastx from BLAST + or Kyoto Encyclopedia of Genes and Genomes (KEGG) Orthology identifier assigned by ghostKOALA is also listed. The percentage of transcripts annotated per category is listed in parentheses.*

### Cluster Enrichment Analysis

A summary of major discussed enrichment categories can be found in Table 3. A full set of GO enrichment results can be found in Supplementary Data Sheets 3, 4, while a full set of KEGG enrichment results can be found in Supplementary Data Sheet 5.

**TABLE 3:** Transcriptional profile cluster enrichment analysis results.

Category	Cluster	Ontology	Description	Example human orthologs
Inflammation	B2/P3	GO BP	Inflammatory response	**ABCC1**, **TNIP1, C5**, BIRC(2,**3**), TLR(3,8), XIAP, **NFKBIA**/BLNK, LYN, PLA2G7
Metabolism	B4/P4	GO BP	Fatty acid metabolic process	**ETFA**, **IVD**, **HADHA**, HPGDS, ACAD(**SB**, 10, S)/APPL2, PCC(A,B), CPT(1A,2)
	B3/B4/P4	ko	Glycolysis/Gluconeogenesis	**PGAM2,** PFKP, PFKL/**PGK1, GAPDH,** ALDOA, **GPI**, **TPI1**/GCK, HK1
	B3/P4	ko	Pyruvate metabolism	**DLD**, PKM, **PDHB**, **ALDH91A**, ME(1,3)/DLAT, MDH(1,2), PDHA2
	B4/P4	ko	Citrate cycle (TCA cycle)	**ACO1**, **ACO2, IDH3A, IDH3B, IDH3G**/IDH1, IDH2, DLST
	B4/P4	ko	Oxidative phosphorylation	NDUF(**A10,B7**,**V1**,**V2**,S2)**, SDHA,** ATP5F1A/SDHD, COX4I1, UQCRC2, TP5M(C3,G,F)
Mitochondria	P4	GO BP	Mitochondrial transport	PPP3R1, SLC25A22, DYNLT1, CPT2, CPT1A, PPIF, IMMP1L
	P4	GO BP	Mitochondrion organization	PRDX3, COA1, GDAP1, SLC25A46, CAMKMT, SOD2, FUNDC1, MPV17, FIS1, TRAK1
Proteostasis	B1/B2	ko	Protein processing in endoplasmic reticulum	DDRGK1, SYVN1, CREB3L3, SEL1L, UGGT1, MANF/ATF4 (CREB2), CREBRF, BCAP31, ANKZF1, EIF2AK3 (PERK), DNAJC3, EEF2
	B2/P1/P2	ko	Lysosome	**CTSL, CTSS, CTSV,** CTSD, LGMN, **PSAP, GM2A**/GALC, CLN5
Ribosome	B2/Pup	ko	Ribosome	**FAU**, RPS(**A**,3,5,7,**12,14,15**, 16,20,21,23,26,**27**), RPL(**P2**,5,7,19,23)
	B2/P1	ko	Aminoacyl-tRNA biosynthesis	(**D,T**,R,C,S,V,K,G,M,F)ARS
	B2/P3	ko	Ribosome biogenesis in eukaryotes	EIF6, NAT10, RPP40, RIOK1, **MDN1, EFL1**/HEATR1, NXT2
Signaling	P3	GO BP	MAPK cascade	TNIP1, BIRC7, MOS, TRIB2, MAPK14, MAP3K8
	P5	GO BP	Presynaptic endocytosis	ITSN1, SYT1, PICALM, ACTB
	B3	GO BP	Regulation of ion transport	CALCR, DYSF, GNB5, NIPSNAP2, PRKACA, PER2, NOS1AP, SYT(4,7,10,15)
	P4	GO BP	Signal release	PARK7, CACNB2, CALM1, STXBP1, SYT7, NCS1, KCNC2, DNAJC5, SNAP29
	B3	GO BP	Synaptic vesicle cycle	STXBP5, ITSN1, PICALM, NRXN1, ERC2, DGKQ, VAMP1, SYN2, DNAJC5
	B3	GO CC	Ion channel complex	GLRA3, KCN(AB2,AB3,C1,J5,N2), CACN(A2D2,A2D3,G7)
	B3/P4	ko	cGMP-PKG signaling pathway	**PRKG1,** PDE2A, GUCY1B1/RHO(A,B,C), VDAC2
	B3/Pdown	ko	Long-term depression	GUCY1B2, **GNAZ, GNAO1, GNAI1**
	B3/P4	ko	Long-term potentiation	**GRIN2(A,B,C), CAMK4, PNCK, NRAS, CALM(1,2,L3), MAP2K(1,3)**/CAMK2D
	P4	ko	Synaptic vesicle cycle	ATP6V1(C1,F,G1), CLTA, NSF, AP2(S1,M1), STXBP1

*A subset of the results is organized into six major representative categories: inflammation, metabolism, mitochondria, proteostasis, ribosome, and signaling. Each row per category lists an ontology term from a specific ontology set (KEGG or GO), the clusters for which it is significantly enriched, and a set of example human orthologs found within each cluster. Terms significantly enriched in multiple separate clusters are separated by “/” with example orthologs from each cluster similarly separated. Orthologs that appear in more than one cluster are in boldface. A complete list of all KEGG and GO enrichments, including p values and other enrichment test metadata, can be found in Supplementary Data Sheets 3–5.*

**FIGURE 5:**
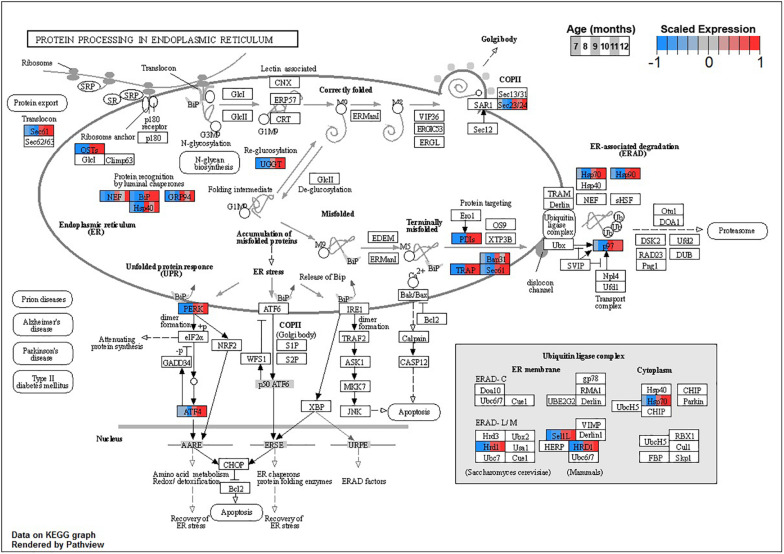
Expression of orthologs annotated for the Kyoto Encyclopedia of Genes and Genomes (KEGG) *protein processing in Endoplasmic Reticulum* (ko04141) pathway present in buccal S sensory neuron (BSC) expression clusters with increasing trend (B1 and B2). Each node of the pathway diagram represents an ortholog assigned to an *Aplysia californica* transcript. Nodes are divided into six colored sections, from left to right, ages 7 to 12 months. Node sections are colored according to their mean centered- and variance scaled-expression, together representing the expression trends of each node across the aging process. Nodes that are not colored were absent from the gene sets of expression clusters B1 and B2. Pathway enrichment demonstrates broad upregulation of proteostatic processes in the endoplasmic reticulum with age.

Enrichment analyses for increasing with age BSC cluster B1 suggest a response to disrupted proteostasis, with significant terms from *clusterProfiler* such as *protein processing in endoplasmic reticulum* (ko04141, Figure 5) and *response to endoplasmic reticulum stress* (GO:0034976). Enrichment from *topGO* further suggests perturbations in proteostasis with terms such as *endoplasmic reticulum quality control compartment* (GO:0044322), as well as some suggestion of inflammation with terms like *regulation of acute inflammatory response* (GO:0002673, Supplementary Data Sheet 3).

Similarly to cluster B1, BSC increasing cluster B2 is enriched for inflammation- and proteostasis-related ontologies with significant terms like *inflammatory response* (GO:0006954) and *lysosome* (ko04142, Table 3). In addition, B2 is enriched for terms relating to ribosomes and protein translation, for example, *ribosome* (ko03010) and *aminoacyl-tRNA biosynthesis* (ko00970, Table 3). Additionally, terms related to the storage of reactive metabolites such as lipid localization (GO:0010876) and iron ion binding (GO:0005506) are notable (Supplementary Data Sheet 3).

**FIGURE 6:**
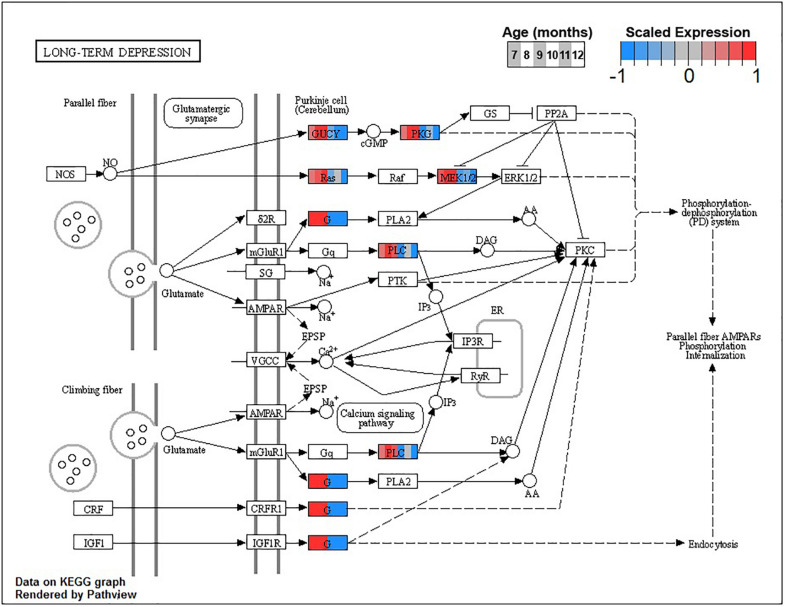
Expression of orthologs annotated for the Kyoto Encyclopedia of Genes and Genomes (KEGG) *long-term depression* (ko04730) pathway present in buccal S sensory neuron (BSC) expression clusters with decreasing trend (B3 and B4). Each node of the pathway diagram represents an ortholog assigned to an *Aplysia californica* transcript. Nodes are divided into six colored sections, from left to right, ages 7 to 12 months. Node sections are colored according to their mean centered and variance scaled expression, together representing the expression trends of each node across the aging process. Nodes that are not colored were absent from the gene sets of expression clusters B3 and B4. Pathway enrichment demonstrates broad downregulation of key signaling cascades in aging.

The larger decreasing BSC cluster, B3, exhibits a large number of enrichments, primarily related to neuronal processes. These include structural components like *ion channel complex* (GO:0034702), synaptic processes such as *synaptic vesicle cycle* (GO:0099504), signaling cascades like *cGMP-PKG signaling pathway* (ko04022), and learning processes including *Long-term potentiation* (ko04720) and *depression* (ko04730, Figure 6 and Table 3).

Finally, monotonically decreasing BSC cluster B4 is enriched primarily for major metabolic processes. These include the major pathways involved in the oxidative metabolism of glucose: *glycolysis/gluconeogenesis* (ko00010), *TCA cycle* (ko00020), and *oxidative phosphorylation* (ko00190, Table 3). Enrichment of terms related to ROS detoxification processes such as *glutathione metabolic process* (GO:0006749) and *ROS metabolic process* (GO:0072593) is also noteworthy (Supplementary Data Sheet 3).

Cluster enrichment of PVC clusters generally reflect similar major categories observed in BSC cluster enrichment (Table 3). The increasing PVC clusters P1, P2, and P3 resemble increasing cluster B2 in their enrichment.

PVC cluster P1 is enriched for proteostatic terms such as *lysosome* (ko04142) *aminoacyl-tRNA biosynthesis* (ko00970, Table 3). In addition, terms related to apoptosis such as cysteine-type endopeptidase inhibitor activity involved in apoptotic process (GO:0043027) are of note (Supplementary Data Sheet 3).

PVC cluster P2 is enriched for terms relating to proteostasis and ribosome such as *lysosome* (ko04142, Table 3) and ribosomal *small subunit biogenesis* (GO:0042274, Supplementary Data Sheet 3). Furthermore, like B2, P2 exhibits enrichment in terms related to volatile metabolite storage such as *lipid storage* (GO:0019915, Supplementary Data Sheet 3) and *mineral absorption* (ko04978, Supplementary Data Sheet 5).

The largest of the three increasing PVC clusters, P3, is similar to cluster B2 in enrichment for ribosome- and inflammation-related terms, such as ribosome biogenesis in eukaryotes (ko03008) and *inflammatory response* (GO:0006954, Table 3). Interestingly, *MAPK cascade* (GO:0000165) is enriched in cluster P3, with many representative orthologs involved in pro-inflammatory signaling, such as MAPK14 (Table 3).

**FIGURE 7:**
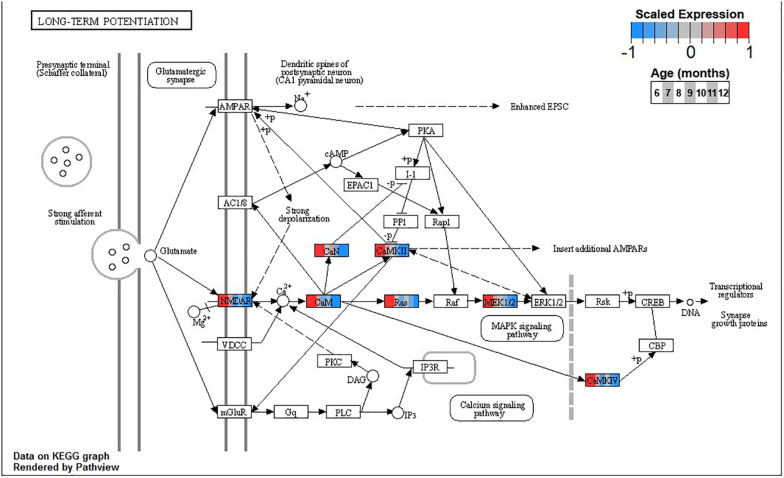
Expression of orthologs annotated for the Kyoto Encyclopedia of Genes and Genomes (KEGG) *long-term potentiation* (ko04720) pathway present in pleural ventral caudal sensory neuron (PVC) expression clusters with decreasing trend (P4 and P5). Each node of the pathway diagram represents an ortholog assigned to an *Aplysia californica* transcript. Nodes are divided into seven colored sections, from left to right, ages 6 to 12 months. Node sections are colored according to their mean centered and variance scaled expression, together representing the expression trends of each node across the aging process. Nodes that are not colored were absent from the gene sets of expression clusters P4 and P5. Pathway enrichment demonstrates broad downregulation of key signaling cascades in aging.

**FIGURE 8:**
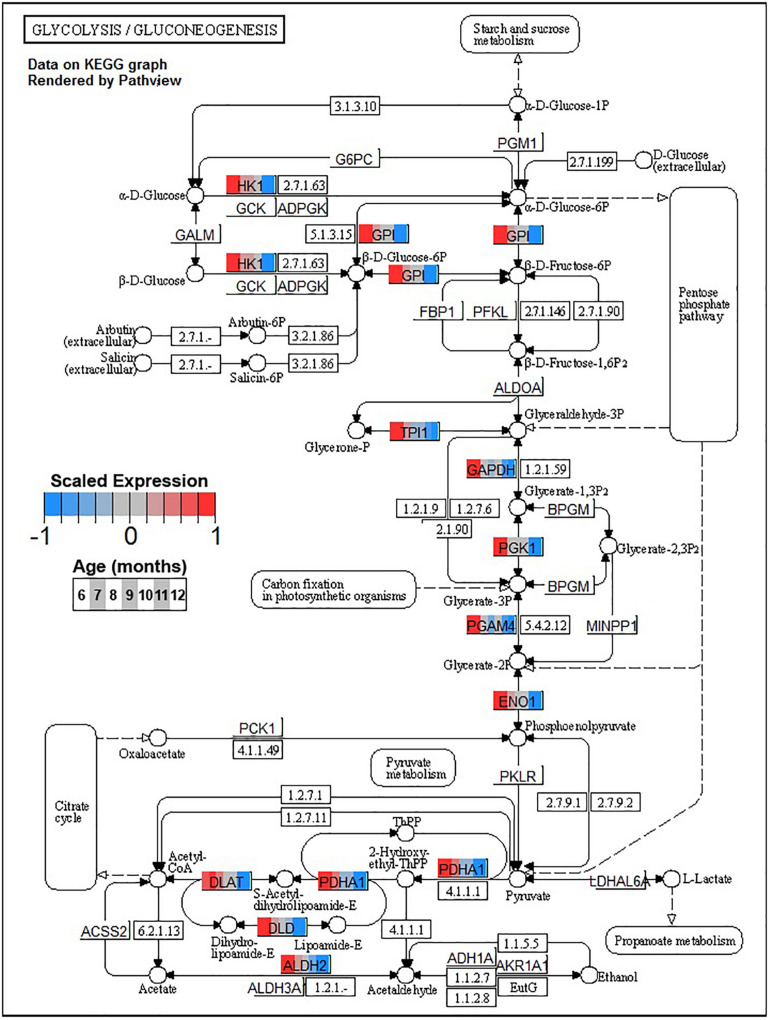
Expression of orthologs annotated for the Kyoto Encyclopedia of Genes and Genomes (KEGG) *glycolysis and gluconeogenesis* (ko00010) pathway present in pleural ventral caudal sensory neuron (PVC) expression clusters with decreasing trend (P4 and P5). Each node of the pathway diagram represents an ortholog assigned to an *Aplysia californica* transcript. Nodes are divided into seven colored sections, from left to right, ages 6 to 12 months. Node sections are colored according to their mean centered and variance scaled expression, together representing the expression trends of each node across the aging process. Nodes that are not colored were absent from the gene sets of expression clusters P4 and P5. Pathway enrichment demonstrates broad downregulation in glucose metabolism with age including pathways involved in synthesis of acetyl-CoA from pyruvate, which feeds into the TCA cycle.

**FIGURE 9:**
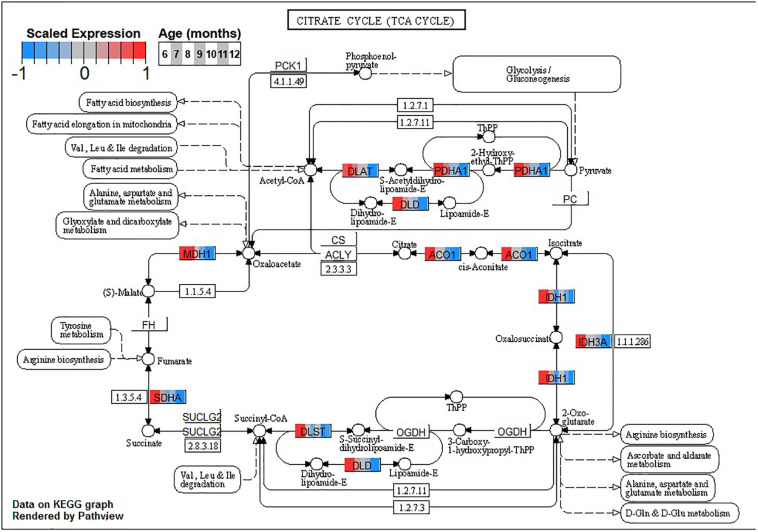
Expression of orthologs annotated for the Kyoto Encyclopedia of Genes and Genomes (KEGG) *tricarboxylic acid cycle* (ko00020) pathway present in pleural ventral caudal sensory neuron (PVC) expression clusters with decreasing trend (P4 and P5). Each node of the pathway diagram represents an ortholog assigned to an *Aplysia californica* transcript. Nodes are divided into seven colored sections, from left to right, ages 6 to 12 months. Node sections are colored according to their mean centered and variance scaled expression, together representing the expression trends of each node across the aging process. Nodes that are not colored were absent from the gene sets of expression clusters P4 and P5. Pathway enrichment suggests decreased oxidation of acetyl-CoA in these neurons with increasing age.

**FIGURE 10:**
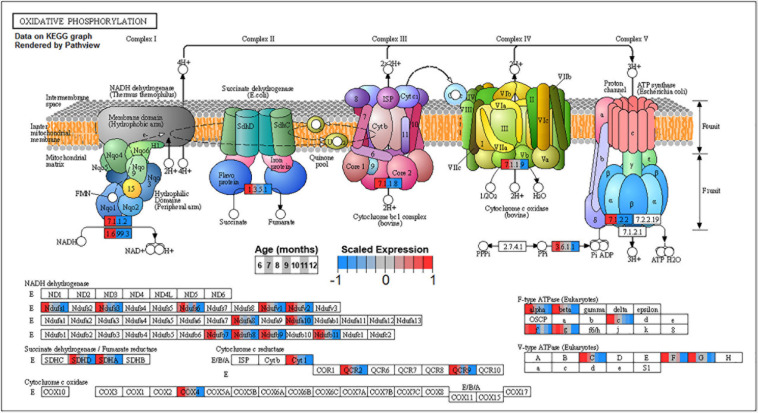
Expression of orthologs annotated for the Kyoto Encyclopedia of Genes and Genomes (KEGG) *oxidative phosphorylation* (ko00190) pathway present in pleural ventral caudal sensory neuron (PVC) expression clusters with decreasing trend (P4 and P5). Each node of the pathway diagram represents an ortholog assigned to an *Aplysia californica* transcript. Nodes are divided into seven colored sections, from left to right, ages 6 to 12 months. Node sections are colored according to their mean centered and variance scaled expression, together representing the expression trends of each node across the aging process. Nodes that are not colored were absent from the gene sets of expression clusters P4 and P5. Pathway enrichment indicates that all components of the electron transport chain in mitochondria are inhibited with aging and thus energy production from ATP synthase is likely to be reduced with aging.

Conversely, enrichment of terms in decreasing PVC cluster P4 resembles an amalgamation of decreasing BSC clusters B3 and B4. P4 is enriched for many of the same terms in cluster B3 associated with neuronal function such as *cGMP-PKG signaling pathway* (ko04022) and *long-term potentiation* (ko04720, Figure 7) or analogous terms like *signal release* (GO:0023061, Table 3). However, P4 is most significantly enriched for the same major metabolic pathways as B4, such as *glycolysis and gluconeogenesis* (ko00010, Figure 8), *TCA cycle* (ko00020, Figure 9), and *oxidative phosphorylation* (ko00190, Figure 10 and Table 3). Interestingly, P4 is further enriched for several processes associated with mitochondrial health, such as *mitochondrion organization* (GO:0007005, Table 3), and antioxidant defense, such as *glutathione metabolism* (ko00480, Supplementary Data Sheet 5). The enrichment of these categories in downregulated cluster P4 together suggests general mitochondrial dysfunction with age.

Finally, decreasing PVC cluster P5 enrichment is sparse but has a similar neuronal character to decreasing clusters B3 and P4. Specifically, P5 is enriched for terms relating to synaptic function, such as *presynaptic endocytosis* (GO:0140238, Table 3) or signaling cascades such as *kinase regulator activity* (GO:0019207, Supplementary Data Sheet 3).

## Discussion

Transcriptional profile clustering of transcripts differentially expressed in aging from Aplysia SN clusters identified four and five coherent transcriptional patterns from BSC and PVC, respectively. GO and KEGG enrichment analysis of these clusters revealed enrichment for energy metabolism, mitochondrial homeostasis, and various signaling pathways in clusters with expression profiles exhibiting decreasing trajectory in aging (clusters B3, B4, P4, and P5). Meanwhile, clusters with increasing transcriptional profile trajectories in aging (clusters B1, B2, P1, P2, and P3) were enriched for pathways associated with inflammation, proteostasis, and ribosomes.

The weight distribution of this cohort was similar to those observed previously in similar rearing conditions (Gerdes and Fieber, 2006; Kempsell and Fieber, 2014). Decreases in weight starting at age 9–10 months is diagnostic of transition into the Aged I life stage. Mortality was similarly within previously reported ranges. Reflex behavior data fell within expected norms and verified that normal aging occurred during late mature and aged stages (Greer et al., 2018). TTR conformed to the Aplysia Stages of Aging model (Kempsell and Fieber, 2014), with 10-month animals representing aged stage AI and 11- to 12-month animals representing stage AII. TWR, despite exhibiting a U-shaped trajectory over the adult lifespan, increased steadily after age 10 months, with TWR times of age 10–12 months corroborating the TTR data, as reported in previous studies (Kempsell and Fieber, 2014; Greer et al., 2018).

PCA analysis and transcriptional trajectories of PVC vs BSC SN suggest that these different neuron types age somewhat differently. Similar phenomena have been described in physiological and transcriptional aging phenotypes between neuron types in Aplysia previously and may echo known brain region-specific aging patterns in humans (Moroz and Kohn, 2010; Kempsell and Fieber, 2014; Marjanska et al., 2017). However, enrichment analysis suggests that aging in these groups of neurons is broadly similar, as many cellular processes known to be affected by aging among other neural aging models are common to both PVC and BSC.

A striking commonality between BSC and PVC aging is the consistent decline in major metabolic pathways with aging as reflected in monotonically decreasing clusters B4 and P4. Age-associated decreases in glucose metabolism have been observed in the nervous systems of flies (Ma et al., 2018) and rodents (Hoyer, 1985) and in several regions of the human brain (Nugent et al., 2014; Camandola and Mattson, 2017). The key enzymes involved in glycolysis are downregulated in both BSC and PVC (Figure 8), indicating overall declines in glucose metabolism (Table 3). Glycolysis has been demonstrated to be particularly important for neurons as the primary fast energy source during metabolically demanding events such as neuronal signaling (Jang et al., 2016; Diaz-Garcia and Yellen, 2019). In mouse, decreases in glycolysis-derived ATP specifically altered synaptic transmission of presynaptic neurons (Lujan et al., 2016), raising the possibility that similar changes in transmission observed in aged PVC previously (Kempsell and Fieber, 2014) may be linked to decreased glycolysis. Clusters B4 and P4 are also enriched for *pyruvate metabolism* (ko00620), suggesting decreased activity of the pyruvate dehydrogenase complex (PDH) and decreased utilization of glucose for Acetyl-CoA flux into the TCA cycle with age.

Decreased activity of PDH and associated shifts toward anaerobic glycolysis has been observed in the aging rat brain (Zhou et al., 2008, 2009). Other sources of Acetyl-CoA for the TCA cycle, namely, *fatty acid metabolic process* (GO:0006631), showed decreases in aging in clusters B4 and P4 as well, suggesting decreased general capacity of the TCA cycle with age in these neurons. Maintenance of Acetyl-CoA levels has been demonstrated to be neuroprotective in aging mouse brains, likely due to sustained TCA cycle activity (Currais et al., 2019). In conjunction with downregulation of input pathways, the TCA cycle itself exhibited many genes with reduced expression in clusters B4 and P4, further suggesting a metabolic shift away from oxidative metabolism of glucose with age.

Decreased TCA activity has also been observed in the aging brains of rodents (Srividhya et al., 2009; Lin et al., 2014), humans (Boumezbeur et al., 2010), and *C. elegans* (Hastings et al., 2019) and in the aging yeast model for post-mitotic cells (Samokhvalov et al., 2004). Decreased activity of the TCA cycle decreases the amount of NADH available for the generation and maintenance of mitochondrial potential, resulting in decreased ATP production, increases in the production ROS such as H_2_O_2_, and impaired NADPH-dependent antioxidant defense (Zhou et al., 2009; Nickel et al., 2015). Decreased TCA activity would suggest decreased activity of the OXPHOS pathway, and indeed clusters B4 and P4 exhibit enrichment for OXPHOS.

The observed downregulation of mitochondrial OXPHOS complex transcripts in PVC and BSC is a phenomenon common to the aging nervous systems of rodents (Zahn et al., 2007; de Magalhaes et al., 2009), human (Glass et al., 2013; Kumar et al., 2013; Mastroeni et al., 2017), worms (McCarroll et al., 2004), flies (Davie et al., 2018), and a short-lived teleost (Baumgart et al., 2014, 2016). These transcriptional changes are known to co-occur with mitochondrial dysfunction that results in decreased ATP production, increased ROS production, and compromised Ca^+2^ buffering capacity (Ojaimi et al., 1999; Ferguson et al., 2005; Pandya et al., 2015, 2016). Neurons and their mitochondria are particularly at risk from the effects of ROS due to their high energy demands and long lifespans (Grimm and Eckert, 2017).

These metabolic impairments contribute to and can be driven by chronic generation of surplus ROS that overwhelm antioxidant defenses and transform otherwise hormetic ROS into drivers of metabolic failure (Grimm and Eckert, 2017; Guo et al., 2020). KEGG and GO enrichment suggests decreased activity of antioxidant systems in both these neuron types. In addition, decreased expression of key antioxidants like *SOD2*, Catalase, and *GPX4* suggests that risk of deleterious ROS damage increases with age in these SN, as observed in other aging models (Sandhu and Kaur, 2002; Haddadi et al., 2014). The decreases in mitochondrial metabolism and antioxidant system transcripts observed suggest that PVC and BSC experience mitochondrial impairment with age, a notion further suggested by downregulation of an ortholog of mitophagy regulator FUNDC1 in both BSC and PVC (Chen et al., 2016), as well as several other mitochondrial maintenance orthologs in PVC. Because mitophagy is a key component in the process of mitochondrial maintenance and recycling, any impairment of this pathway would likely exacerbate mitochondrial dysfunction and the resultant loss of energy metabolism and ROS management in aging cells.

The aforementioned shift from aerobic to anaerobic use of glucose-derived pyruvate due to decreased PHD activity in aging rat brain is hypothesized to be in response to increased H_2_O_2_ production in NADH-depleted mitochondria (Zhou et al., 2008, 2009). Furthermore, oxidative damage has been shown to impair activity of numerous TCA enzymes, such as isocitrate dehydrogenase and aconitase, in many aging models (Das et al., 2001; Schriner et al., 2005; Di Domenico et al., 2010; Hastings et al., 2019; Guo et al., 2020). Both of these enzymes are also downregulated in PVC and BSC with age (Figure 9). Increases in brain oxidation state is a known hallmark of brain aging observed across species, and KEGG and GO enrichment results suggest that PVC and BSC exhibit an increased oxidative state in aging as well (Garaschuk et al., 2018). In this state, ROS compromises the function of not only metabolic enzymes (Di Domenico et al., 2010) but also proteins key to neuronal function such as calcium sensors and neurotransmitter receptors, contributing to age-associated cognitive impairment (Gao et al., 1998; Pieta Dias et al., 2007; Haxaire et al., 2012). Under normal conditions, protein oxidization is mitigated with antioxidant systems and oxidized proteins are efficiently removed by lysosomes and proteasomes (Hohn et al., 2013; Jackson and Hewitt, 2016). However, in aging, these antioxidant and proteolytic systems are known to become inefficient and neurons experience proteostatic stress (Chaudhari et al., 2014).

KEGG and GO enrichment analyses suggest that proteostasis is compromised in both tissues in aging. Particularly in BSC, upregulation with age in endoplasmic reticulum protein folding-related transcripts in monotonically increasing cluster B1 presages the sharp inflection in cluster B2 that is enriched for further ER-based proteostatic mechanisms. BSC cluster B2 is further enriched for the lysosome KEGG pathways, which plays a key role in proteostasis by breaking down misfolded proteins (Jackson and Hewitt, 2016). Similarly, cluster P2, which inflects at sexual maturity and then increases monotonically with age, is also enriched for the lysosome pathway. Furthermore, BSC and, to a lesser extent, PVC show upregulations for key members of the ER stress signaling pathway, namely, homologs of *EIF2AK3* (*PERK*) and *ATF4* (*CREB2*) with age. This suggests that disruption of proteostasis begins early and remains a persistent and mounting threat in aging, as observed in *Drosophila* (Yang et al., 2019).

In post-mitotic and long lived cells, such as Aplysia neurons, lysosomes accumulate aggregates of oxidized protein called lipofuscin with age (Papka et al., 1981; Terman and Brunk, 2004). This process is accelerated by mitochondrial dysfunction (Konig et al., 2017). Indigestible lipofuscin decreases lysosome efficiency and scavenges proteolytic enzymes from autophagosomes, allowing for persistence of malfunctioning mitochondria (Kurz et al., 2008). An age-associated decrease in lysosome efficiency could be particularly problematic for neurons that rely on lysosomes to facilitate synaptic remodeling (Kononenko, 2017). Accumulation of lipofuscins has been demonstrated to increase lysosome number in cultured fibroblasts (Hohn et al., 2012). A similar response may explain enrichment of lysosomal proteins in increasing clusters in both BSC and PVC. Enrichment of ribosome and protein synthesis processes in clusters upregulated in aging, a phenomenon also observed in the short-lived teleost *Nothobranchius furzeri*, may reflect this increasing demand for lysosomes and activated endoplasmic reticulum stress response in the face of proteostatic stress (Baumgart et al., 2014). In addition to oxidized proteins, lipofuscin is also composed of accumulated metals and oxidized lipids.

Enrichment analysis in clusters B2 and P2 suggests that both PVC and BSC also accumulate lipids and iron with age. Iron levels in the brain increase steadily with age in rodent and human (Dedman et al., 1992), causing increased expression of the iron storage proteins ferritin and neuro-melanin (Zecca et al., 2001; Bartzokis et al., 2004; Walker et al., 2016). Both BSC and PVC exhibited an age-dependent upregulation in homologs of iron storage protein and aging biomarker Ferritin Heavy Chain (*FTH1*). Although this process is known to occur in healthy aging, increased iron is considered a risk factor for and contributor to several age-associated neurodegenerative diseases (Ndayisaba et al., 2019).

BSC and PVC also exhibited age-dependent upregulation in a homolog for lipid storage droplet (LD) biomarker Perilipin 2 (*PLIN2*). Accumulation of LD in non-adipose tissues, including neurons, as a result of aging has been described in humans, worms, rodents, and Aplysia (Savage et al., 1987; Conte et al., 2016; Shimabukuro et al., 2016; Palikaras et al., 2017). Lipid sequestration into LD functions to protect oxidation susceptible lipids from ROS. Oxidative stress as a result of mitochondrial dysfunction and/or iron accumulation is known to induce this formation of LD in the brain and foreshadows the onset of age-associated neurodegenerative disease (Campos et al., 2015; Liu et al., 2015). In the pond snail *Lymnaea stagnalis*, a gastropod relative of Aplysia, oxidation of membrane lipids has been shown to drive age-associated decreases in neuronal excitability (Hermann et al., 2014). Therefore, the increased lipid oxidation rate suggested by increased *PLIN2* expression may be responsible for similar decreases in neuronal excitability and conduction velocity in aging observed in Aplysia previously (Kempsell and Fieber, 2014, 2016).

In both BSC and PVC, increases in lysosome-related transcripts as well as lipid and iron storage biomarkers co-occurred in the same cluster, suggesting a response to a common underlying driver. In PVC, this mirrors the linear decrease of mitochondrial metabolism cluster P4. In BSC, meanwhile, this occurs in cluster B2, which exhibits a strong negative inflection that lags behind the decrease of mitochondrial metabolism cluster B4. Overall, these patterns suggest a common response to declines in neuronal metabolism. Lipofuscin formation, iron sequestration, and LD formation all occur as a result of and contribute to ROS accumulation, which is known to be a result of mitochondrial dysfunction. These data suggest that metabolic impairment may be the driver of these aging hallmarks, as suggested in the Energy Maintenance Theory of Aging (Chaudhari and Kipreos, 2018) and the Free-Radical-Induced Energetic and Neural Decline in Senescence (FRIENDS) theory of aging (Raz and Daugherty, 2018).

Decreases in metabolic activity in aging may also compromise the energetically expensive signaling functions of these SN. Indeed, pathways and processes crucial to normal neuronal signaling, such as synaptic release of neurotransmitter and synaptic remodeling during potentiation and depression, were downregulated in aging along with major metabolic pathways. In BSC, decreased expression in major metabolic pathways in cluster B4 temporally preceded the strong negative inflection in cluster B3, which is enriched primarily in neuronal signaling pathways. This may suggest that, in BSC, not only are metabolic and signaling pathways related, as in PVC, but that it is the decline in energy metabolism that drives decreases in normal neuronal function. Decreased glycolysis in particular, as suggested by enrichment of decreasing clusters, may adversely affect the ability of these SN to transmit incoming sensory input across the synapse (Jang et al., 2016; Lujan et al., 2016; Diaz-Garcia and Yellen, 2019). Furthermore, knock-on effects of compromised TCA and OXPHOS activity, namely, compromised Ca^+2^ signaling due to impaired mitochondria and ER stress, as well as compromised functioning of signaling pathways and neurotransmitter receptors due to ROS damage, likely also contribute to impaired neuronal function.

Decreasing expression of genes associated with long-term potentiation in clusters B3 and P4 suggests that the chemical mechanisms underpinning simple learning are impaired with age in these SN, specifically through decreases in plasma membrane receptors, calcium sensing via calmodulin, and the MAPK signaling cascade. During strong stimulation to elicit long-term facilitation (LTF), such as five pulses of 5HT, protein kinase A (PKA) stimulates secretion of autocrine peptide sensorin, which, in turn, activates and translocates MAPK to the nucleus (Hu et al., 2004). There, MAPK removes CREB2 transcriptional repression and phosphorylates CREB1 and C/EBP, thus mediating transcriptional events necessary for LTF (Martin et al., 1997; Michael et al., 1998). Inhibition of MAPK or its activator MAPK/ERK kinase (MAP2K1, MEK) blocks LTF. Both MEK and its upstream activator and calmodulin target Ras are downregulated in BSC and PVC, suggesting impairment of the MAPK signaling cascade needed for induction of long-term memory in aging. This may contribute to previously observed decreases in 5-HT induced facilitation in PVC with age (Kempsell and Fieber, 2015a). Interestingly, while the MAPK cascade is downregulated in both BSC and PVC, *CREB2* (*ATF4*) is upregulated, likely due to proteostatic stress, suggesting that the response to disruptions in proteostasis may be connected to observed impairment in learning with age.

In addition to PKA, protein kinase G (PKG) may also play an important role in compromised learning in aging observed in BSC and PVC. In mammals, noxious stimulation of nociceptive neurons activates PKG that promotes MAPK translocation to the nucleus and induces long-term hyperexcitability (LTH) (Komatsu et al., 2009). Similarly, pinch stimulus to SN in Aplysia activates the PKG signaling pathway and results in activation and translocation of MAPK, resulting in LTH (Lewin and Walters, 1999; Sung et al., 2004). In addition to the MAPK cascade, the *cGMP-PKG signaling pathway* (ko04022), including PKG specifically, is significantly decreased in clusters B3 and P4 with aging. Similarly, long-term operant memory in learning that a food item is inedible previously has been shown to depend on PKG-mediated MAPK signaling in Aplysia, raising the possibility that the age-related memory impairments observed in BSC and PVC may be due to decreased PKG expression (Michel et al., 2011; Kempsell and Fieber, 2015b). Given the role of NO-derived PKG signaling in the feeding behavior of Aplysia (Susswein and Chiel, 2012), observed downregulation of PKG with age may also contribute to observed decreased feeding and resultant weight loss observed in aged Alysia (Fieber et al., 2010).

Together, these data suggest that decreases in oxidative metabolism precede later hallmarks of neuronal aging. Initial declines in glucose metabolism result in decreased energy available for homeostatic functions, such as protein folding and ROS detoxification. A feedback loop ensues, in which increased mitochondrial dysfunction induces more oxidative stress. Further impaired metabolism continues until a threshold is reached and the native antioxidant and proteostatic defenses are no longer sufficient. In response, neurons transition from antioxidant defense to damage control by sequestering volatile iron with ferritins, packaging ROS susceptible lipids into LD with perilipins, and accumulating lipofuscins in lysosomes. Metabolic impairment and increasing resource expenditure on sequestration and repair diverts limited energy reserves away from neuronal functions such as restoration of resting membrane potential and other facets of synaptic transmission, which disrupts normal signaling. Impaired signaling results in cognitive impairment at the whole organism level. Thus, age-associated cognitive impairment ultimately arises from metabolic declines in relevant neurons.

Although these age-associated changes in Aplysia SN were studied in the context of non-pathological aging, aging and age-associated disease exist on a continuum (Franceschi et al., 2018). Indeed, many of the described changes are common underlying symptoms of both aging and age-associated neurodegenerative disease. Metabolic compromise, particularly of OXPHOS, and associated mitochondrial dysfunction are classical hallmarks of Alzheimer’s and Parkinson’s disease. Similarly, oxidative stress as a result of mitochondrial compromise-derived ROS is common to the etiologies of many neurodegenerative diseases, including Alzheimer’s, Parkinson’s, and beyond (Kim et al., 2015; Islam, 2017). Impaired proteostasis defines many neurodegenerative diseases, with accumulation of aggregates of malformed proteins being defining diagnostic features for many, such as α-synuclein for Parkinson’s and amyloid-β and tau for Alzheimer’s (Hetz and Mollereau, 2014; Kurtishi et al., 2019). Furthermore, as mentioned earlier, the accumulation of iron and LD in neurons is a risk factor and an early indicator of the onset of neurodegenerative disease (Bartzokis et al., 2004; Silvestri and Camaschella, 2008; Liu and Connor, 2012; Ward et al., 2014; Liu et al., 2015; Han et al., 2018). These data suggest that Aplysia SN exhibit transcriptional signatures of these same processes. Effective investigation of age-associated neurodegenerative disease requires study in the context in which they arise: the aging nervous system (Johnson, 2015; Wallace and Howlett, 2016). These results demonstrate that Aplysia SN present an excellent model system for doing just that.

## Data Availability Statement

The datasets presented in this study can be found in online repositories. The names of the repository/repositories and accession number(s) can be found below: https://www.ncbi.nlm.nih.gov/, PRJNA639857.

## Author Contributions

NK and LF designed this study with input from MS. NK collected data, executed the experiment, and analyzed the data with input from LF and MS. NK wrote the manuscript with input from LF and MS. All authors read and approved the final manuscript.

## Conflict of Interest

The authors declare that the research was conducted in the absence of any commercial or financial relationships that could be construed as a potential conflict of interest.

## Footnotes

1 http://www.bioinformatics.bbsrc.ac.uk/projects/fastqc

2 https://ftp.ncbi.nlm.nih.gov/genomes/all/annotation_releases/6500/101/GCF_000002075.1_AplCal3.0/
