# Supplementary material for: Changes in Metabolism and Proteostasis Drive Aging Phenotype in Aplysia californica Sensory Neurons
Source: Front Aging Neurosci. 2020 Sep 15;12:573764. doi: 10.3389/fnagi.2020.573764 (PMC7522570; doi:10.3389/fnagi.2020.573764)
Supplement: Supplementary file 1 [file Table_1.DOCX]

**Supplementary Table 1.** All Software and respective versions used for RNA sequencing read quality control and quality assurance, mapping and abundance estimation, and downstream analysis. A further list of software packages used in the R statistical environment is available in Supplementary Table 2.

| Software | Version | link |
| --- | --- | --- |
| FastQC | 0.10.1 | https://www.bioinformatics.babraham.ac.uk/projects/fastqc/ |
| BBTools | 37.90 | https://jgi.doe.gov/data-and-tools/bbtools/ |
| Salmon | 0.11.2 | https://combine-lab.github.io/salmon/ |
| R | 3.6.0 | https://www.r-project.org/ |
| RStudio | 1.2.1335 | https://rstudio.com/ |
