# Supplementary material for: Changes in Metabolism and Proteostasis Drive Aging Phenotype in Aplysia californica Sensory Neurons
Source: Front Aging Neurosci. 2020 Sep 15;12:573764. doi: 10.3389/fnagi.2020.573764 (PMC7522570; doi:10.3389/fnagi.2020.573764)
Supplement: Supplementary file 8 [file Data_Sheet_1.PDF]

Supplementary Data Sheet 1. Annotation completeness of Kyoto Encyclopedia of Genes and Genomes pathways from orthology annotation of *Aplysia californica* RefSeq proteome by ghostKOALA service.

Module Reconstruction Result

Show all objects

Pathway module

Carbohydrate metabolism

Central carbohydrate metabolism

M00001 Glycolysis (Embden-Meyerhof pathway), glucose => pyruvate [PATH:map00010 map01200 map01100] (44) (complete)
M00002 Glycolysis, core module involving three-carbon compounds [PATH:map00010 map01200 map01230 map01100] (19) (complete)
M00003 Gluconeogenesis, oxaloacetate => fructose-6P [PATH:map00010 map00020 map01100] (22) (complete)
M00307 Pyruvate oxidation, pyruvate => acetyl-CoA [PATH:map00010 map00020 map00620 map01200 map01100] (6) (complete)
M00009 Citrate cycle (TCA cycle, Krebs cycle) [PATH:map00020 map01200 map01100] (31) (complete)
M00010 Citrate cycle, first carbon oxidation, oxaloacetate => 2-oxoglutarate [PATH:map00020 map01200 map01210 map01230 map01100] (10) (complete)
M00011 Citrate cycle, second carbon oxidation, 2-oxoglutarate => oxaloacetate [PATH:map00020 map01200 map01100] (21) (complete)
M00004 Pentose phosphate pathway (Pentose phosphate cycle) [PATH:map00030 map01200 map01100 map01120] (17) (complete)
M00006 Pentose phosphate pathway, oxidative phase, glucose 6P => ribulose 5P [PATH:map00030 map01200 map01100 map01120] (8) (complete)
M00007 Pentose phosphate pathway, non-oxidative phase, fructose 6P => ribose 5P [PATH:map00030 map01200 map01230 map01100 map01120] (7) (complete)
M00580 Pentose phosphate pathway, archaea, fructose 6P => ribose 5P [PATH:map00030 map01200 map01230 map01100 map01120] (2) (1 block missing)
M00005 PRPP biosynthesis, ribose 5P => PRPP [PATH:map00030 map00230 map01200 map01230 map01100] (1) (complete)
M00008 Entner-Doudoroff pathway, glucose-6P => glyceraldehyde-3P + pyruvate [PATH:map00030 map01200 map01100 map01120] (6) (2 blocks missing)

Other carbohydrate metabolism

M00014 Glucuronate pathway (uronate pathway) [PATH:map00040 map01100] (36) (1 block missing)
M00632 Galactose degradation, Leloir pathway, galactose => alpha-D-glucose-1P [PATH:map00052 map01100] (11) (complete)
M00129 Ascorbate biosynthesis, animals, glucose-1P => ascorbate [PATH:map00040 map00053 map01100] (26) (1 block missing)
M00854 Glycogen biosynthesis, glucose-1P => glycogen/starch [PATH:map00500 map01100] (8) (complete)
M00855 Glycogen degradation, glycogen => glucose-6P [PATH:map00500 map01100] (6) (complete)
M00549 Nucleotide sugar biosynthesis, glucose => UDP-glucose [PATH:map00520 map01100] (16) (complete)
M00554 Nucleotide sugar biosynthesis, galactose => UDP-galactose [PATH:map00520 map00052 map01100] (2) (complete)
M00012 Glyoxylate cycle [PATH:map00630 map01200 map01100 map01110] (6) (2 blocks missing)
M00013 Malonate semialdehyde pathway, propanoyl-CoA => acetyl-CoA [PATH:map00410 map00640 map01200 map01100] (12) (1 block missing)
M00741 Propanoyl-CoA metabolism, propanoyl-CoA => succinyl-CoA [PATH:map00280 map00630 map00640 map01200 map01100] (7) (complete)
M00130 Inositol phosphate metabolism, PI=> PIP2 => Ins(1,4,5)P3 => Ins(1,3,4,5)P4 [PATH:map00562 map01100] (19) (complete)
M00131 Inositol phosphate metabolism, Ins(1,3,4,5)P4 => Ins(1,3,4)P3 => myo-inositol [PATH:map00562 map01100] (15) (complete)
M00132 Inositol phosphate metabolism, Ins(1,3,4)P3 => phytate [PATH:map00562 map01100] (4) (complete)

Energy metabolism

Carbon fixation

M00166 Reductive pentose phosphate cycle, ribulose-5P => glyceraldehyde-3P [PATH:map00710 map01200 map01100 map01120] (3) (2 blocks missing)
M00167 Reductive pentose phosphate cycle, glyceraldehyde-3P => ribulose-5P [PATH:map00710 map01200 map01100 map01120] (11) (1 block missing)

M00168 CAM (Crassulacean acid metabolism), dark [PATH:map00620 map00710 map01200 map01100 map01120] (3) (1 block missing)
M00169 CAM (Crassulacean acid metabolism), light [PATH:map00620 map00710 map01200 map01100 map01120] (2) (1 block missing)
M00170 C4-dicarboxylic acid cycle, phosphoenolpyruvate carboxykinase type [PATH:map00710 map01200 map01100 map01120] (2) (2 blocks missing)
Methane metabolism
M00345 Formaldehyde assimilation, ribulose monophosphate pathway [PATH:map00030 map00680 map01200 map01230 map01120] (4) (2 blocks missing)
M00344 Formaldehyde assimilation, xylulose monophosphate pathway [PATH:map00680 map01200 map01120] (2) (2 blocks missing)
Sulfur metabolism
M00176 Assimilatory sulfate reduction, sulfate => H2S [PATH:map00920 map01100 map01120] (1) (2 blocks missing)

Lipid metabolism

Fatty acid biosynthesis and degradation
M00082 Fatty acid biosynthesis, initiation [PATH:map00061 map01212 map01100] (20) (complete)
M00083 Fatty acid biosynthesis, elongation [PATH:map00061 map01212 map01100] (22) (complete)
M00085 Fatty acid biosynthesis, elongation, mitochondria [PATH:map00062 map01212 map01100] (7) (complete)
M00415 Fatty acid biosynthesis, elongation, endoplasmic reticulum [PATH:map00062 map01040 map01212 map01100] (26) (complete)
M00086 beta-Oxidation, acyl-CoA synthesis [PATH:map00061 map00071 map01212 map01100] (14) (complete)
M00087 beta-Oxidation [PATH:map00071 map01212 map01100] (18) (complete)
M00861 beta-Oxidation, peroxisome, VLCFA [PATH:map01040 map01212] (10) (complete)
Sterol biosynthesis
M00101 Cholesterol biosynthesis, squalene 2,3-epoxide => cholesterol [PATH:map00100 map01100] (13) (complete)
M00103 Cholecalciferol biosynthesis [PATH:map00100 map01100] (2) (1 block missing)
M00106 Conjugated bile acid biosynthesis, cholate => taurocholate/glycocholate [PATH:map00120 map01100] (4) (1 block missing)
M00862 beta-Oxidation, peroxisome, tri/dihydroxycholestanoyl-CoA => choloyl/chenodeoxycholoyl-CoA [PATH:map00120 map01100] (3) (1 block missing)
M00107 Steroid hormone biosynthesis, cholesterol => prognenolone => progesterone [PATH:map00140 map01100] (1) (1 block missing)
M00110 C19/C18-Steroid hormone biosynthesis, pregnenolone => androstenedione => estrone [PATH:map00140 map01100] (21) (2 blocks missing)
Lipid metabolism
M00088 Ketone body biosynthesis, acetyl-CoA => acetoacetate/3-hydroxybutyrate/acetone [PATH:map00072 map01100] (12) (1 block missing)
M00089 Triacylglycerol biosynthesis [PATH:map00561 map01100] (25) (complete)
M00098 Acylglycerol degradation [PATH:map00561 map01100] (13) (complete)
M00090 Phosphatidylcholine (PC) biosynthesis, choline => PC [PATH:map00564 map01100] (3) (complete)
M00092 Phosphatidylethanolamine (PE) biosynthesis, ethanolamine => PE [PATH:map00564 map01100] (10) (complete)
M00093 Phosphatidylethanolamine (PE) biosynthesis, PA => PS => PE [PATH:map00564 map01100] (1) (2 blocks missing)
M00094 Ceramide biosynthesis [PATH:map00600 map01100] (8) (complete)
M00066 Lactosylceramide biosynthesis [PATH:map00600 map01100] (3) (1 block missing)
M00067 Sulfoglycolipids biosynthesis, ceramide/1-alkyl-2-acylglycerol => sulfatide/seminolipid [PATH:map00600 map00565 map01100] (3) (complete)
M00099 Sphingosine biosynthesis [PATH:map00600 map01100] (10) (complete)
M00100 Sphingosine degradation [PATH:map00600 map01100] (3) (complete)

Nucleotide metabolism

Purine metabolism
M00048 Inosine monophosphate biosynthesis, PRPP + glutamine => IMP [PATH:map00230 map01100] (17) (complete)
M00049 Adenine ribonucleotide biosynthesis, IMP => ADP,ATP [PATH:map00230 map01100] (18) (complete)
M00050 Guanine ribonucleotide biosynthesis IMP => GDP,GTP [PATH:map00230 map01100] (17) (complete)
Pyrimidine metabolism
M00051 Uridine monophosphate biosynthesis, glutamine (+ PRPP) => UMP [PATH:map00240

map01100] (9) (complete)
M00052 Pyrimidine ribonucleotide biosynthesis, UMP => UDP/UTP,CDP/CTP [PATH:map00240 map01100] (9) (complete)
M00053 Pyrimidine deoxyribonuleotide biosynthesis, CDP/CTP => dCDP/dCTP,dTDP/dTTP [PATH:map00240 map01100] (14) (2 blocks missing)
M00046 Pyrimidine degradation, uracil => beta-alanine, thymine => 3-aminoisobutanoate [PATH:map00240 map01100] (4) (complete)

Amino acid metabolism

Serine and threonine metabolism
M00020 Serine biosynthesis, glycerate-3P => serine [PATH:map00260 map00680 map01200 map01230 map01100] (3) (complete)
M00555 Betaine biosynthesis, choline => betaine [PATH:map00260 map01100] (1) (1 block missing)

Cysteine and methionine metabolism
M00021 Cysteine biosynthesis, serine => cysteine [PATH:map00270 map00920 map01200 map01230 map01100 map01110] (1) (1 block missing)
M00338 Cysteine biosynthesis, homocysteine + serine => cysteine [PATH:map00260 map00270 map01230 map01100] (5) (complete)
M00034 Methionine salvage pathway [PATH:map00270 map01100] (15) (complete)
M00035 Methionine degradation [PATH:map00270 map01100] (12) (complete)
M00368 Ethylene biosynthesis, methionine => ethylene [PATH:map00270 map01110] (3) (2 blocks missing)

Branched-chain amino acid metabolism
M00036 Leucine degradation, leucine => acetoacetate + acetyl-CoA [PATH:map00280 map01100] (18) (complete)

Lysine metabolism
M00032 Lysine degradation, lysine => saccharopine => acetoacetyl-CoA [PATH:map00310 map01100] (18) (complete)

Arginine and proline metabolism
M00844 Arginine biosynthesis, ornithine => arginine [PATH:map00220 map01230 map01100] (7) (complete)
M00029 Urea cycle [PATH:map00220 map01230 map01100] (7) (2 blocks missing)
M00015 Proline biosynthesis, glutamate => proline [PATH:map00330 map01230 map01100] (6) (complete)
M00047 Creatine pathway [PATH:map00330 map01100] (1) (2 blocks missing)

Polyamine biosynthesis
M00133 Polyamine biosynthesis, arginine => agmatine => putrescine => spermidine [PATH:map00330 map01100] (4) (2 blocks missing)
M00134 Polyamine biosynthesis, arginine => ornithine => putrescine [PATH:map00330 map01100] (3) (1 block missing)
M00135 GABA biosynthesis, eukaryotes, putrescine => GABA [PATH:map00330 map01100] (16) (complete)

Histidine metabolism
M00045 Histidine degradation, histidine => N-formiminoglutamate => glutamate [PATH:map00340 map01100] (8) (complete)

Aromatic amino acid metabolism
M00025 Tyrosine biosynthesis, chorismate => tyrosine [PATH:map00400 map01230 map01100] (1) (1 block missing)
M00042 Catecholamine biosynthesis, tyrosine => dopamine => noradrenaline => adrenaline [PATH:map00350 map01100] (8) (1 block missing)
M00044 Tyrosine degradation, tyrosine => homogentisate [PATH:map00350 map01100] (6) (1 block missing)
M00037 Melatonin biosynthesis, tryptophan => serotonin => melatonin [PATH:map00380 map01100] (6) (1 block missing)
M00038 Tryptophan metabolism, tryptophan => kynurenine => 2-aminomuconate [PATH:map00380 map01100] (14) (1 block missing)

Other amino acid metabolism
M00027 GABA (gamma-Aminobutyrate) shunt [PATH:map00250 map00650 map01100] (7) (complete)
M00118 Glutathione biosynthesis, glutamate => glutathione [PATH:map00480 map01100] (9) (complete)

Glycan metabolism

Glycan biosynthesis
M00055 N-glycan precursor biosynthesis [PATH:map00510 map01100] (18) (complete)
M00072 N-glycosylation by oligosaccharyltransferase [PATH:map00510 map00513 map01100] (10) (complete)
M00073 N-glycan precursor trimming [PATH:map00510 map01100] (8) (complete)

M00056 O-glycan biosynthesis, mucin type core [PATH:map00512 map01100] (40) (1 block missing)
M00065 GPI-anchor biosynthesis, core oligosaccharide [PATH:map00563 map01100] (10) (complete)
M00070 Glycosphingolipid biosynthesis, lacto-series, LacCer => Lc4Cer [PATH:map00601 map01100] (24) (1 block missing)
M00071 Glycosphingolipid biosynthesis, neolacto-series, LacCer => nLc4Cer [PATH:map00601 map01100] (5) (1 block missing)

Glycosaminoglycan metabolism

M00057 Glycosaminoglycan biosynthesis, linkage tetrasaccharide [PATH:map00532 map00534 map01100] (4) (complete)
M00058 Glycosaminoglycan biosynthesis, chondroitin sulfate backbone [PATH:map00532 map01100] (12) (complete)
M00059 Glycosaminoglycan biosynthesis, heparan sulfate backbone [PATH:map00534 map01100] (7) (complete)
M00076 Dermatan sulfate degradation [PATH:map00531 map01100] (25) (complete)
M00077 Chondroitin sulfate degradation [PATH:map00531 map01100] (24) (complete)
M00078 Heparan sulfate degradation [PATH:map00531 map01100] (32) (complete)
M00079 Keratan sulfate degradation [PATH:map00531 map01100] (20) (complete)

Metabolism of cofactors and vitamins

Cofactor and vitamin metabolism

M00115 NAD biosynthesis, aspartate => NAD [PATH:map00760 map01100] (4) (2 blocks missing)
M00120 Coenzyme A biosynthesis, pantothenate => CoA [PATH:map00770 map01100] (14) (complete)
M00126 Tetrahydrofolate biosynthesis, GTP => THF [PATH:map00790 map00670 map01100] (8) (2 blocks missing)
M00842 Tetrahydrobiopterin biosynthesis, GTP => BH4 [PATH:map00790 map01100] (6) (complete)
M00843 L-threo-Tetrahydrobiopterin biosynthesis, GTP => L-threo-BH4 [PATH:map00790 map01100] (5) (1 block missing)
M00140 C1-unit interconversion, prokaryotes [PATH:map00670 map01100] (3) (2 blocks missing)
M00141 C1-unit interconversion, eukaryotes [PATH:map00670 map01100] (7) (complete)
M00868 Heme biosynthesis, animals and fungi, glycine => heme [PATH:map00860] (22) (complete)
M00121 Heme biosynthesis, plants and bacteria, glutamate => heme [PATH:map00860 map01100 map01110] (18) (2 blocks missing)
M00128 Ubiquinone biosynthesis, eukaryotes, 4-hydroxybenzoate => ubiquinone [PATH:map00130 map01100] (10) (complete)

Biosynthesis of terpenoids and polyketides

Terpenoid backbone biosynthesis

M00095 C5 isoprenoid biosynthesis, mevalonate pathway [PATH:map00900 map01100 map01110] (11) (complete)
M00849 C5 isoprenoid biosynthesis, mevalonate pathway, archaea [PATH:map00900 map01110 map01130] (9) (2 blocks missing)
M00364 C10-C20 isoprenoid biosynthesis, bacteria [PATH:map00900 map01100 map01110 map01130] (4) (complete)
M00365 C10-C20 isoprenoid biosynthesis, archaea [PATH:map00900 map01100 map01110] (3) (1 block missing)
M00366 C10-C20 isoprenoid biosynthesis, plants [PATH:map00900 map01100 map01110] (5) (1 block missing)
M00367 C10-C20 isoprenoid biosynthesis, non-plant eukaryotes [PATH:map00900 map01100 map01110] (6) (complete)

Structural complex

Energy metabolism

ATP synthesis

M00142 NADH:ubiquinone oxidoreductase, mitochondria [PATH:map00190] (6) (complete)
M00143 NADH dehydrogenase (ubiquinone) Fe-S protein/flavoprotein complex, mitochondria [PATH:map00190] (11) (complete)
M00146 NADH dehydrogenase (ubiquinone) 1 alpha subcomplex [PATH:map00190] (13) (1 block missing)
M00147 NADH dehydrogenase (ubiquinone) 1 beta subcomplex [PATH:map00190] (11) (1 block missing)
M00148 Succinate dehydrogenase (ubiquinone) [PATH:map00190] (6) (complete)
M00151 Cytochrome bc1 complex respiratory unit [PATH:map00190] (3) (complete)
M00152 Cytochrome bc1 complex [PATH:map00190] (9) (1 block missing)
M00154 Cytochrome c oxidase [PATH:map00190] (16) (1 block missing)
M00158 F-type ATPase, eukaryotes [PATH:map00190] (16) (1 block missing)

M00160 V-type ATPase, eukaryotes [PATH:map00190] (23) (complete)

Genetic information processing

DNA polymerase

M00261 DNA polymerase alpha / primase complex [PATH:map03030] [BR:ko03032] (7) (complete)
M00262 DNA polymerase delta complex [PATH:map03030 map03410 map03420] [BR:ko03032 ko03400] (5) (complete)
M00263 DNA polymerase epsilon complex [PATH:map03030] [BR:ko03032 ko03400] (5) (1 block missing)
M00293 DNA polymerase zeta complex [PATH:map03460] [BR:ko03400] (6) (complete)
M00294 DNA polymerase gamma complex [BR:ko03032] (3) (complete)

Replication system

M00284 Origin recognition complex [PATH:map04110 map04111 map04113] [BR:ko03032] (11) (complete)
M00285 MCM complex [PATH:map03030 map04110 map04111 map04113] [BR:ko03032] (7) (complete)
M00286 GINS complex [BR:ko03032] (6) (complete)
M00288 RPA complex [PATH:map03030 map03420 map03430 map03440] [BR:ko03032 ko03400] (5) (1 block missing)
M00289 RF-C complex [PATH:map03030 map03420 map03430] [BR:ko03032 ko03400] (9) (complete)
M00424 Shelterin complex [BR:ko03032] (4) (1 block missing)

Repair system

M00296 BER complex [PATH:map03410] [BR:ko03400] (20) (complete)
M00290 Holo-TFIIH complex [PATH:map03420] [BR:ko03021 ko03400] (9) (complete)
M00291 MRN complex [PATH:map03440] [BR:ko03032 ko03400] (6) (complete)
M00292 MRX complex [PATH:map03440 map03450] [BR:ko03400] (5) (1 block missing)
M00297 DNA-PK complex [PATH:map03450 map04110] [BR:ko03032 ko03400] (3) (complete)
M00295 BRCA1-associated genome surveillance complex (BASC) [PATH:map03430 map03440 map03460] [BR:ko03400] (27) (complete)
M00413 FA core complex [PATH:map03460] [BR:ko03400] (14) (1 block missing)
M00414 Bloom's syndrome complex [PATH:map03460] [BR:ko03400] (9) (complete)

RNA polymerase

M00180 RNA polymerase II, eukaryotes [PATH:map03020 map05016] [BR:ko03021 ko03400] (14) (1 block missing)
M00181 RNA polymerase III, eukaryotes [PATH:map03020 map04623] [BR:ko03021] (21) (complete)
M00182 RNA polymerase I, eukaryotes [PATH:map03020] [BR:ko03021] (11) (1 block missing)

Spliceosome

M00351 Spliceosome, U1-snRNP [PATH:map03040] [BR:ko03041] (9) (complete)
M00352 Spliceosome, U2-snRNP [PATH:map03040] [BR:ko03041] (24) (complete)
M00354 Spliceosome, U4/U6.U5 tri-snRNP [PATH:map03040] [BR:ko03041] (41) (complete)
M00355 Spliceosome, 35S U5-snRNP [PATH:map03040] [BR:ko03041] (43) (complete)
M00353 Spliceosome, Prp19/CDC5L complex [PATH:map03040] [BR:ko03041] (15) (complete)
M00396 Lsm 2-8 complex [PATH:map03040 map03018] [BR:ko03041] (10) (complete)
M00397 Lsm 1-7 complex [PATH:map03018] (9) (complete)
M00398 Sm core complex [PATH:map03040] [BR:ko03032 ko03041] (7) (complete)
M00399 Cap binding complex [PATH:map03013 map03015 map03040 map03040] [BR:ko03041] (2) (complete)

RNA processing

M00425 H/ACA ribonucleoprotein complex [PATH:map03008] [BR:ko03032 ko03009] (3) (1 block missing)
M00426 Survival motor neuron (SMN) complex [PATH:map03013] [BR:ko03041] (8) (complete)
M00427 Nuclear pore complex [PATH:map03013] (55) (complete)
M00428 eIF4F complex [PATH:map03013] [BR:ko03012] (15) (complete)
M00430 Exon junction complex (EJC) [PATH:map03013 map03015 map03040] [BR:ko03041] (30) (complete)
M00405 THC complex [PATH:map03040 map03013] [BR:ko03041] (5) (complete)
M00406 TREX complex [PATH:map03013 map03040] [BR:ko03041] (8) (complete)
M00390 Exosome, archaea [PATH:map03018] (4) (complete)
M00391 Exosome, eukaryotes [PATH:map03018] (10) (complete)
M00392 Ski complex [PATH:map03018] (5) (complete)
M00393 TRAMP complex [PATH:map03018] (5) (complete)
M00394 RNA degradosome [PATH:map03018] (4) (1 block missing)
M00395 Decapping complex [PATH:map03018] (8) (complete)

Ribosome

M00178 Ribosome, bacteria [PATH:map03010] [BR:ko03011] (54) (1 block missing)
M00179 Ribosome, archaea [PATH:map03010] [BR:ko03011] (78) (1 block missing)

M00177 Ribosome, eukaryotes [PATH:map03010] [BR:ko03011] (91) (complete)
Proteasome
M00340 Proteasome, 20S core particle [PATH:map03050] [BR:ko03051] (16) (complete)
M00341 Proteasome, 19S regulatory particle (PA700) [PATH:map03050] [BR:ko03051] (20) (complete)
M00337 Immunoproteasome [PATH:map03050] [BR:ko03051] (13) (1 block missing)
Ubiquitin system
M00379 SCF-MET30 complex [PATH:map04120 map04111] [BR:ko04121] (4) (1 block missing)
M00380 SCF-BTRC complex [PATH:map04120 map04114 map04310 map04710] [BR:ko04121] (6) (complete)
M00381 SCF-SKP2 complex [PATH:map04120 map04110 map05168] [BR:ko04121] (9) (complete)
M00382 SCF-FBS complex [PATH:map04120 map04141] [BR:ko04121] (4) (1 block missing)
M00407 SCF-CDC4 complex [PATH:map04120 map04111] [BR:ko04121] (4) (1 block missing)
M00411 SCF-GRR1 complex [PATH:map04120 map04111] [BR:ko04121] (4) (1 block missing)
M00383 ECV complex [PATH:map04120 map05211] [BR:ko04121] (8) (complete)
M00384 Cul3-SPOP complex [PATH:map04120] [BR:ko04121] (5) (complete)
M00385 Cul4-DDB1-DDB2 complex [PATH:map04120 map03420] [BR:ko04121 ko03400] (8) (complete)
M00386 Cul4-DDB1-CSA complex [PATH:map04120 map03420] [BR:ko04121 ko03400] (6) (complete)
M00387 SCF-FBW7 complex [PATH:map04120] [BR:ko04121] (10) (complete)
M00388 ECS complex [PATH:map04120] [BR:ko04121] (16) (complete)
M00389 APC/C complex [PATH:map04120 map04110 map04114 map04914 map04111 map04113] [BR:ko04121 ko03036] (19) (1 block missing)
Protein processing
M00400 p97-Ufd1-Npl4 complex [PATH:map04141] (5) (complete)
M00401 Sec61 complex [PATH:map03060 map04141 map04145 map05110] [BR:ko02044] (2) (1 block missing)
M00402 Translocon-associated protein (TRAP) complex [PATH:map04141] (4) (complete)
M00403 HRD1/SEL1 ERAD complex [PATH:map04141] (13) (1 block missing)
M00404 COPII complex [PATH:map04141] (11) (complete)
M00408 ESCRT-0 complex [PATH:map04144] (5) (complete)
M00409 ESCRT-I complex [PATH:map04144] (9) (complete)
M00410 ESCRT-II complex [PATH:map04144] (3) (complete)
M00412 ESCRT-III complex [PATH:map04144] (16) (complete)

Environmental information processing

Phosphate and amino acid transport system
M00222 Phosphate transport system [PATH:map02010] [BR:ko02000] (2) (1 block missing)
Peptide and nickel transport system
M00239 Peptides/nickel transport system [PATH:map02010] [BR:ko02000] (1) (1 block missing)
Bacterial secretion system
M00335 Sec (secretion) system [PATH:map03070] [BR:ko02044] (4) (1 block missing)
M00429 Competence-related DNA transformation transporter [BR:ko02044] (1) (1 block missing)

Functional set

Metabolism

Aminoacyl tRNA
M00360 Aminoacyl-tRNA biosynthesis, prokaryotes [PATH:map00970] (67) (complete)
M00359 Aminoacyl-tRNA biosynthesis, eukaryotes [PATH:map00970] (69) (complete)
Nucleotide sugar
M00362 Nucleotide sugar biosynthesis, prokaryotes [PATH:map00520] (16) (1 block missing)
M00361 Nucleotide sugar biosynthesis, eukaryotes [PATH:map00520] (16) (complete)

Environmental information processing

Cell signaling
M00687 MAPK (ERK1/2) signaling [PATH:map04010 map04013 map04011] (7) (complete)
M00688 MAPK (JNK) signaling [PATH:map04010 map04013] (26) (complete)
M00689 MAPK (p38) signaling [PATH:map04010 map04013] (24) (complete)
M00690 MAPK (ERK5) signaling [PATH:map04010] (3) (complete)
M00677 Wnt signaling [PATH:map04310] (31) (complete)
M00682 Notch signaling [PATH:map04330] (39) (complete)
M00678 Hedgehog signaling [PATH:map04340 map05217] (6) (2 blocks missing)
M00679 BMP signaling [PATH:map04350 map04390 map04550] (16) (1 block missing)
M00683 Hippo signaling [PATH:map04390 map04391] (12) (complete)
M00684 JAK-STAT signaling [PATH:map04630] (13) (complete)
M00695 cAMP signaling [PATH:map04024] (15) (complete)
M00694 cGMP signaling [PATH:map04022] (30) (complete)

M00676 PI3K-Akt signaling [PATH:map04151 map04068 map04910] (10) (complete)
M00691 DNA damage-induced cell cycle checkpoints [PATH:map04110] (5) (1 block missing)
M00692 Cell cycle - G1/S transition [PATH:map04110 map04111] (16) (complete)
M00693 Cell cycle - G2/M transition [PATH:map04110 map04111] (5) (1 block missing)
M00685 Apoptotic machinery [PATH:map04210 map04214 map04215] (6) (2 blocks missing)
M00686 Toll-like receptor signaling [PATH:map04620 map04064] (22) (2 blocks missing)
