# Supplementary material for: Changes in Metabolism and Proteostasis Drive Aging Phenotype in Aplysia californica Sensory Neurons
Source: Front Aging Neurosci. 2020 Sep 15;12:573764. doi: 10.3389/fnagi.2020.573764 (PMC7522570; doi:10.3389/fnagi.2020.573764)
Supplement: Supplementary file 13 [file Data_Sheet_6.PDF]

GhostKOALA

Result

Your GhostKOALA job Help

Query dataset: 27591 entries
KEGG database searched: c_family_euk+genus_prok+viruses
Job submitted: Thu Mar 7 07:12:02 JST 2019
Job completed: Thu Mar 7 14:17:16 JST 2019

Annotation data Preview first 100 | Download

Summary 13441 entries (48.7%) annotated

Functional category

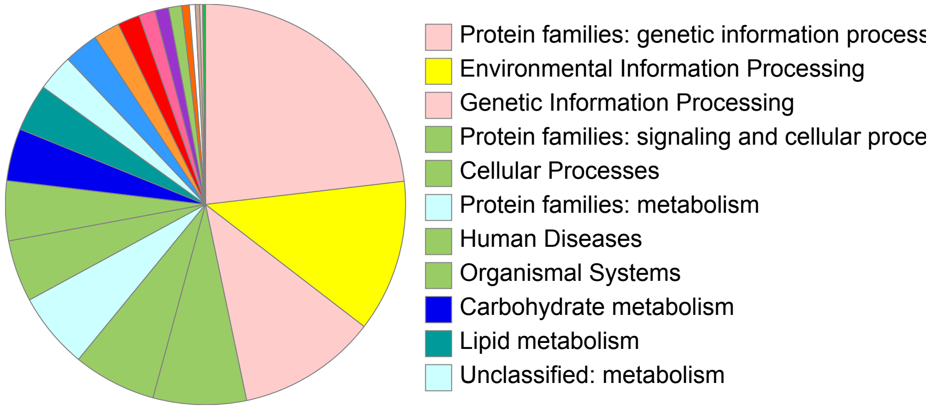

See color codes

KEGG Mapper Reconstruct Pathway
Reconstruct Brite
Reconstruct Module

Taxonomy data Preview first 100 | Download

Taxonomic category

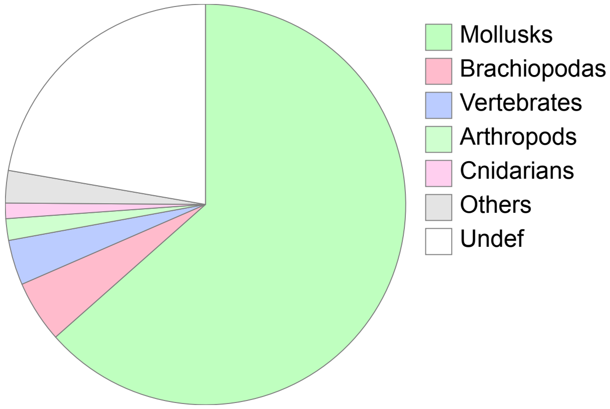

More details View all taxonomic categories (and perform KEGG Mapper analysis)

[GhostKOALA | KEGG | Kanehisa Labs]

Supplementary Figure 1. Results of Kyoto Encyclopedia of Genes and Genomes orthology annotation of *Aplysia californica* RefSeq proteome by ghostKOALA service.
